# Identification of Orbivirus Non-Structural Protein 5 (NS5), Its Role and Interaction with RNA/DNA in Infected Cells

**DOI:** 10.3390/ijms24076845

**Published:** 2023-04-06

**Authors:** Fauziah Mohd Jaafar, Baptiste Monsion, Peter P. C. Mertens, Houssam Attoui

**Affiliations:** 1 UMR1161 VIROLOGIE, INRAE, Ecole Nationale Vétérinaire d’Alfort, ANSES, Université Paris-Est, F-94700 Maisons-Alfort, France; faojaafar@gmail.com (F.M.J.); baptiste.monsion@vet-alfort.fr (B.M.); 2 One Virology, The Wolfson Centre for Global Virus Research, School of Veterinary Medicine and Science, University of Nottingham, Sutton Bonington Campus, Leicestershire LE12 5RD, UK; peter.mertens@nottingham.ac.uk

**Keywords:** bluetongue, bluetongue virus, orbivirus, African horsesickness virus, Kemerovo virus, AHSV, BTV, KEMV, NS5

## Abstract

Bioinformatic analyses have predicted that orbiviruses encode an additional, small non-structural protein (NS5) from a secondary open reading frame on genome segment 10. However, this protein has not previously been detected in infected mammalian or insect cells. NS5-specific antibodies were generated in mice and were used to identify NS5 synthesised in orbivirus-infected BSR cells or cells transfected with NS5 expression plasmids. Confocal microscopy shows that although NS5 accumulates in the nucleus, particularly in the nucleolus, which becomes disrupted, it also appears in the cell cytoplasm, co-localising with mitochondria. NS5 helps to prevent the degradation of ribosomal RNAs during infection and reduces host-cell protein synthesis However, it helps to extend cell viability by supporting viral protein synthesis and virus replication. Pulldown studies showed that NS5 binds to ssRNAs and supercoiled DNAs and demonstrates interactions with ZBP1, suggesting that it modulates host-cell responses.

## 1. Introduction

The International Committee on Taxonomy of Viruses (ICTV) has officially recognised twenty-two virus species within the genus *Orbivirus*, one of six genera classified within the family *Sedoroviridae* (order *Reovirales*), although some recent orbivirus isolates/strains may represent additional distinct species [1,2]. The orbiviruses are arthropod-borne and transmitted between susceptible hosts by the bite of “vector-competent” hematophagous arthropods, including *Culicoides* midges, ticks, phlebotomine flies, and anopheline or culicine mosquitoes, in which they also replicate [3,4]. Collectively, the orbiviruses have a wide host range that includes ruminants, equids, humans, marsupials, and other mammals, as well as reptiles and birds [3,4,5]. Three of the *Orbivirus* species include economically important viruses that can infect livestock species: *Bluetongue virus* (BTV) (the *Orbivirus* “type–species”), *African horse sickness virus* (AHSV), and *Epizootic haemorrhagic disease virus* (EHDV), which are transmitted by *Culicoides* biting midges.

The orbivirus genome consists of ten segments of linear double-stranded RNA (dsRNA). Initial coding assignments for the BTV genome segments identified seven structural proteins (VP1–VP7) and three non-structural proteins (NS1–NS3) [6]. The BTV core particle is composed of two concentric protein layers: the inner “sub-core” layer (containing 120 copies of the T2 protein [VP3 of BTV] per particle), surrounded by the “core-surface” layer (composed of 780 copies of the T13 protein [VP7 of BTV] per particle) [7,8]. Three minor enzymatic proteins (VP1 (Pol), an RNA-dependent RNA polymerase; VP4 (Cap), an RNA capping enzyme; and VP6 (Hel), a helicase) constitute multiple transcription complexes associated with the ten dsRNA genome segments located within the internal lumen of the core [9,10,11]. Two structural proteins (VP2 and VP5 of BTV) form an outer-capsid layer, mediating cell attachment and penetration, respectively, during the initiation of infection [5,12]. The larger outer-capsid protein (VP2 of BTV, EHDV, or AHSV) induces a protective immune response in the vertebrate host, interacting with neutralising antibodies and controlling virus serotype within each orbivirus species [13].

Four non-structural (NS) proteins (NS1–NS4) encoded by the orbivirus genome have previously been detected in infected cells [14,15]. The most abundant of these is NS1, which forms characteristic “tubules” within the cytoplasm of infected cells [16] and is believed to facilitate the movement of newly formed virus particles to the cell membrane [17,18] and acts as a positive regulator of viral mRNA translation [19]. NS2 is the main constituent of viral inclusion bodies, which represent the main site of virus genome replication and progeny particle assembly [20,21]. NS3 and NS3a are membrane-embedded glycoproteins that facilitate virus egress by budding from infected cells, playing a role in vector competence and virulence [22,23].

Most orbivirus genome segments code for only one protein, translated in each case from a single large open reading frame (ORF), although segments 9 and 10 code for two nearly identical proteins in each case (VP6 and VP6a from Seg-9; NS3 and NS3a from Seg-10) [17,24]. The smaller proteins, VP6a or NS3a, are generated by the initiation of translation from second in-phase AUG codons close to the upstream termini. However, bioinformatic analyses have identified additional, small ORFs that overlap the “main” ORF in orbivirus genome segments 9 and 10 [25,26]. The existence of the additional non-structural protein NS4, encoded by Seg-9, has been confirmed by expression and serological studies [14,15]. NS4 exhibits significant sequence and size diversity between the different orbivirus species [14,25] and is believed to counter the innate immune response, representing an additional virulence determinant [15].

Initial observations have indicated potential functions for another predicted translation product from a small down-stream ORF on Seg-10, provisionally identified as NS5 [27]. However, the existence of this protein and its potential for interaction with cellular proteins have not been demonstrated directly by its detection in infected cells [27]. We report the identification and functional characterisation of this fifth orbivirus non-structural protein (NS5, encoded by genome Seg-10) of both insect- and tick-borne orbiviruses.

## 2. Results

### 2.1. Bioinformatics Analyses

BTV-1 and BTV-8 NS5 (59 aa) and BTV-26 NS5 (50 aa) were predicted to contain monopartite and bipartite nuclear localisation signals (NLS) using the programme cNLS Mapper, as shown in Table 1. NS5 of AHSV-4 (83 aa) and EHDV-7Is (50 aa) were only predicted to contain monopartite NLSs, while the KEMV NS5 sequence (62 aa) was not predicted to contain any NLSs.

Proteins containing six residue poly-arginine stretches can usually accumulate in the nucleolar compartment, which is more acidic than the rest of the nucleus, although four successive arginines can be sufficient within the context of an NLS [28]. The NS5 of BTV-8 does contain four successive arginine residues and may therefore localise to nucleoli, although these residues are not found in the NS5 of BTV-1, AHSV-4, or EHDV-7, which were therefore considered unlikely to localise to that compartment.

**Table 1:** The programme cNLS Mapper [29] was used to predict monopartite (a) and bipartite (b) nuclear localisation signals (NLS) in the amino acid (aa) sequences of NS5.

(a)			
Virus	aa Start Position	Monopartite NLS Sequence	Score
BTV-1	37	VLRKHKKRRRL	12.5
	38	LRKHKKRRRLH	13.5
	39	RKHKKRRRLH	6
	39	RKHKKRRRLHS	12
BTV-8	37	VQRSHKRRRRL	12
	39	RSHKRRRRLH	12
BTV-26	28	VLRKHKKRRRL	12.5
	29	LRKHKKRRRLH	13.5
	30	RKHKKRRRLH	6
	30	RKHKKRRRLHS	12
AHSV-4	50	GRLKMKKQRLVLW	6.5
EHDV-7Is	28	VQPWHKKLRRL	6
KEMV		None	
**(b)**			
**Virus**	**aa Start Position**	**Bipartite NLS Sequence**	**Score**
BTV-1	12	RVRLCHRRCLLLHLKSWTKQCQIQRVLRKHKKR	7.3
BTV-8	26	RSWIKRCQIQRVQRSHKRRRR	12.5
	36	RVQRSHKRRRRLHSHRTQKRFVM	9
BTV-26	5	RQRRRRCRLLPSKFWTKRCQTQLVLRKHKKR	7.7
	5	RQRRRRCRLLPSKFWTKRCQTQLVLRKHKKR	6.6
	17	KFWTKRCQTQLVLRKHKKRRR	14
	17	KFWTKRCQTQLVLRKHKKRRRL	13.1
AHSV-4		None	
EHDV-7Is		None	
KEMV		None	

Proteins that exclusively localise to the nucleus score > 8, proteins partially localising to the nucleus score between 6–8, and proteins that theoretically localise to both the nucleus and the cytoplasm score between 3 and 5, while a score below 2 is attributed to cytoplasmic proteins.

**Figure 1:**
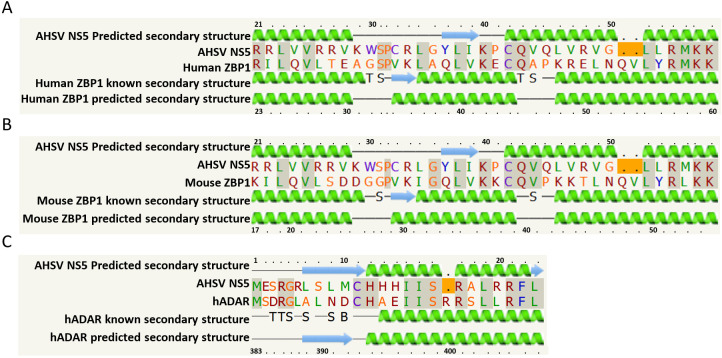
Secondary structure predictions for the amino acid sequence of AHSV NS5 (KP939687 (AHSV-4), KP939916.1 (AHSV-6), KT030659.1 (AHSV-8), and KP940226.1 (AHSV-9)). In the known structures of human ZBP1 (**A**), mouse ZBP1 (**B**), or hADAR (**C**), blue arrows indicate beta sheets and green coils indicate alpha helices; T is a hydrogen-bonded turn; B is a residue in an isolated β-bridge and S is a bend.

Analysis of NS5 sequences using the Phyre-2 programme strongly indicated homologies with nucleic acid binding proteins, including transcription factors. In particular, similarities were detected with Z-alpha protein domains, including those of the human Z-DNA/RNA-binding protein 1 (ZBP1) and dsRNA interacting proteins (e.g., ADAR2), with confidence levels of ~30%. The secondary structure predictions made by Phyre-2 are shown in Figure 1.

**Figure 2:**
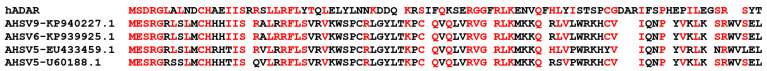
Alignment of the hADAR sequence (aa 1–92) with selected sequences of AHSV NS5. Conserved motifs/residues in the aligned sequences are shown in red.

The predicted secondary structure of AHSV NS5 (aa 21–56) is similar to that of human ZBP1 (aa 23–60) or mouse ZBP1 (aa 17–54). ZBP1 is a sensor involved in a range of pathways, including the innate immune response and PANoptosis [30]. The secondary structure of aa 1–24 of AHSV NS5 was also predicted to be similar to aa 383–407 of human ADAR (hADAR). Alignment of the hADAR sequence (aa 1–92) with the full-length sequence of NS5 showed conserved motifs/positions (Figure 2). ADAR (dsRNA-specific adenosine deaminase) is an enzyme that is involved in RNA editing.

**Figure 3:**
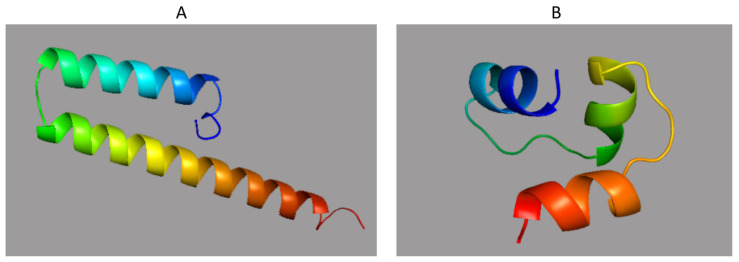
Structural models for NS5 of BTV (**A**) and AHSV (**B**). BTV NS5’s secondary structure is mainly predicted to be helical, while that of AHSV NS5 is typical of the winged tri-helical bundle structure of proteins that interact with Z-forms of nucleic acids.

The 3D structure of BTV-1, predicted by the RaptorX programme, is shown in Figure 3. The protein is predicted to adopt a mainly helical structure with an RMSD of 2.5 A° (RMSD: root-mean-square deviation measures the average distance between backbone atoms of superimposed proteins). Phyre2 predicted the structure of AHSV NS5 (Figure 3) as a winged tri-helical bundle similar to the Z-alpha domain (aa 6–74) of ZBP1. Certain transcriptional repressors that also bind Z-forms of nucleic acids have a similar fold (winged tri-helical bundle), including the PefI protein (70 aa long, accession Q04822) and [Fe-S]-dependent transcriptional repressor (79 aa long, accession B5XTS6.1) [31]. The PBP2 (PolB1 Binding Protein 2, 70 aa long, accession 5N35_1) subunit of the archeal DNA polymerase holoenzyme also has a winged tri-helical bundle fold/structure [31], although this subunit stimulates rather than represses transcription. Additional proteins with a similar fold include the bacterial dissimilatory sulfite reductase (DsvD, 78 aa long, accession Q46582.1), the *Sulfolobus islandicus* protein Sul7s (59 aa long, accession 7BZH_1), and a putative DNA-binding protein of *Cupriavidus pinatubonensis* (95 aa long, accession Q46TT3). It should be noted that the role of these three proteins in nucleic acid binding has only been inferred by their similarity [31,32].

**Figure 4:**
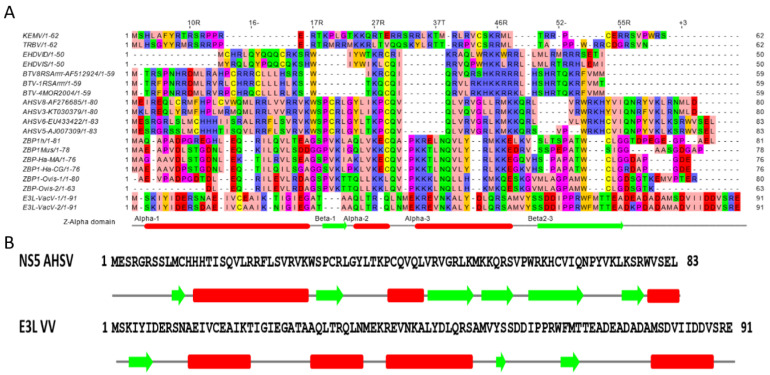
Prediction of secondary structures of orbivirus NS5 proteins and their relatedness to the Z-alpha domain of mammalian ZBP1 proteins and vaccinia virus E3L. Amino acid sequences were aligned with the help of the Clustal × programme. Predictions were made in JPred (**A**) and predictprotein (**B**). Amino acids are coloured according to their physicochemical properties: residues ILVAM (aliphatic hydrophobic: pink), FWY (aromatic: orange), KRH (positively charged: purple), DE (negatively charged: red), STNQ (hydrophilic: bright green), PG (conformationally special: magenta), and C (cysteine: yellow). The alignment in (**A**) shows that the various proteins have conserved residues/motifs, which is consistent with a Z-alpha domain organization. Arrows represent sequences predicted as beta sheets, and bars represent sequences predicted as alpha helices. In (**B**), amino acids 1–83 (full-length) of AHSV NS5 and 1–91 (Z-alpha domain) of VV E3L were subjected to secondary structure predictions. A comparison of the two predicted secondary structural organisations of NS5 and E3L indicates that the two proteins contain certain positionally conserved beta sheets and alpha helices.

Representative sequences of the NS5 of BTV, AHSV, EHDV, KEMV, and TRBV (Tribec virus) were aligned with the sequences of the Z-alpha domain of ZBP1 (human, mouse, hamster, equine, and ovine), E3L of vaccinia virus and ADAR as shown in Figure 4A. Globally, these proteins have been predicted to contain a Z-alpha domain, which suggests that NS5 likely interacts with nucleic acids.

Previous analyses of the human/mouse ZBP1 or vaccinia E3L Z-alpha domains indicated an alpha1-beta1-alpha2-alpha3-beta2-beta3 fold [33] (Figure S1). The secondary structure of BTV NS5, predicted using Jpred and predictprotein (www.predictprotein.org, accessed on 22 January 2019), includes a long helical stretch between aa 17 and 51, with beta sheets towards the beginning and end of the sequence (Figure S2). These analyses indicate an NS5 domain with a similar organisation to Z-alpha of E3L of vaccinia virus and a fold for AHSV NS5 that is globally similar to E3L (Figure 4B and Figure S3), again suggesting that NS5 is likely to interact with nucleic acids.

The shortest orbivirus genome segment (Seg-10) encodes both NS3 and the smaller NS5 protein. The genome of rotavirus A (RVA) encodes 8 structural proteins (VP1 to VP8) and 6 non-structural proteins (NSP1 to NSP6), with NSP5 and NSP6 encoded by overlapping ORFs on the smallest segment, Seg-11 [34]. Although NSP6 (91–95 amino acids in length) is predicted to be mainly alpha helical in structure (Figure S4), it has a similar size to AHSV NS5s (74–83 amino acids). Sequence comparisons of RVA NSP6 and AHSV NS5 suggest that these two proteins are likely orthologues (Figures S4 and S5), and previous studies have suggested that NSP6 of RVA localises to the mitochondria [35]. Therefore, we used iMLP programme (a predictor for internal matrix targeting-like sequences in mitochondrial proteins) analyses to assess whether NS5 of various orbiviruses contains internal matrix targeting signal-like sequences (iMTS-Ls). The significant and similar iMLP scores generated for NS5 of BTV, AHSV, EHDV, and KEMV suggest that they do contain iMTS-Ls (with the highest score attributed to NS5 of BTV and AHSV) (Figure S6). The same programme also predicted that NSP6 of RVA contains iMTS-Ls with similar scores to those of KEMV or EHDV NS5 (Figure S6). In comparison, other proteins such as BTV NS3 or VP6 do not appear to contain iMTS-Ls (Figure S6). These data suggested that NS5 of orbiviruses may localise to mitochondria.

### 2.2. Western Blot Analysis of BTV-1-Infected BSR or KC Cell Lysates, Using Anti-Sera Raised against GST-NS5 Expressed in Bacteria

NS5 of BTV-1, BTV-8, AHSV-4, and KEMV were expressed as GST-fused proteins in bacteria. Although insoluble when expressed at 37 °C, these fusion proteins were recovered in a soluble form at 28 °C and were subsequently purified by glutathione affinity chromatography (Figure S7). However, attempts to keep NS5 in a soluble form after cleavage of the fusion protein using 3C protease were unsuccessful. The soluble GST-fused NS5 of BTV, AHSV, or KEMV were therefore used in both biochemical analysis and to raise antibodies in mice.

**Figure 5:**
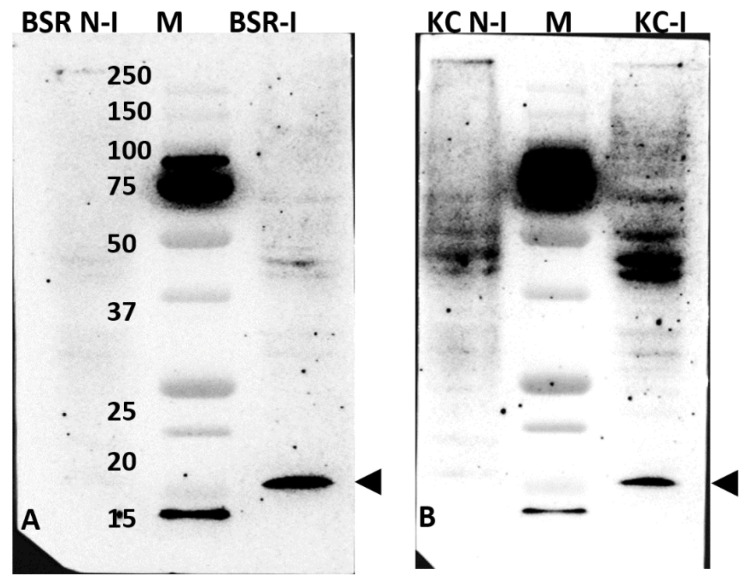
Western blot analysis of BTV-1-infected BSR (left panel) or KC (left panel) cell lysates. Mouse anti-NS5 antiserum was initially adsorbed onto non-infected BSR cells to remove non-specific antibodies. Non-infected and BTV-1-infected BSR (**A**) or KC (**B**) cell lysates were probed using the pre-adsorbed anti-sera, showing a protein band in infected lysates at ~15 KDa (as indicated by the arrowhead). This band was not detected in the non-infected lysates. M = size markers.

Western blotting of BSR or KC cell lysates infected with BTV-1, using murine anti-serum raised against the GST-fused NS5 of BTV, identified in both cases a band with an apparent molecular weight of about 15 kDa that was not present in non-infected cells (Figure 5). This indicates the presence of NS5 specific antibodies in the sera. Bands of ~15 kDa of His-tagged BTV or AHSV NS5 were also detected in pulldown assays of lysates prepared from BSR cells transfected with mammalian expression plasmid pCI-BTV1NS5-6xHis (Figure S8). However, the theoretical molecular weight of BTV NS5 is ~7.5 kDa, suggesting that NS5 may have a secondary structure that reduces its rate of migration during PAGE or may be subject to post-translational modification, increasing its apparent molecular weight. In our previous studies, we also found that BTV NS4 had a higher apparent molecular weight of ~17 kDa in infected cells (as detected by PAGE), despite having a much lower theoretical molecular weight of ~9.5 kDa [14].

### 2.3. Detection of NS5 in Infected Cells by Fluorescence Microscopy

#### 2.3.1. BTV NS5

**Figure 6:**
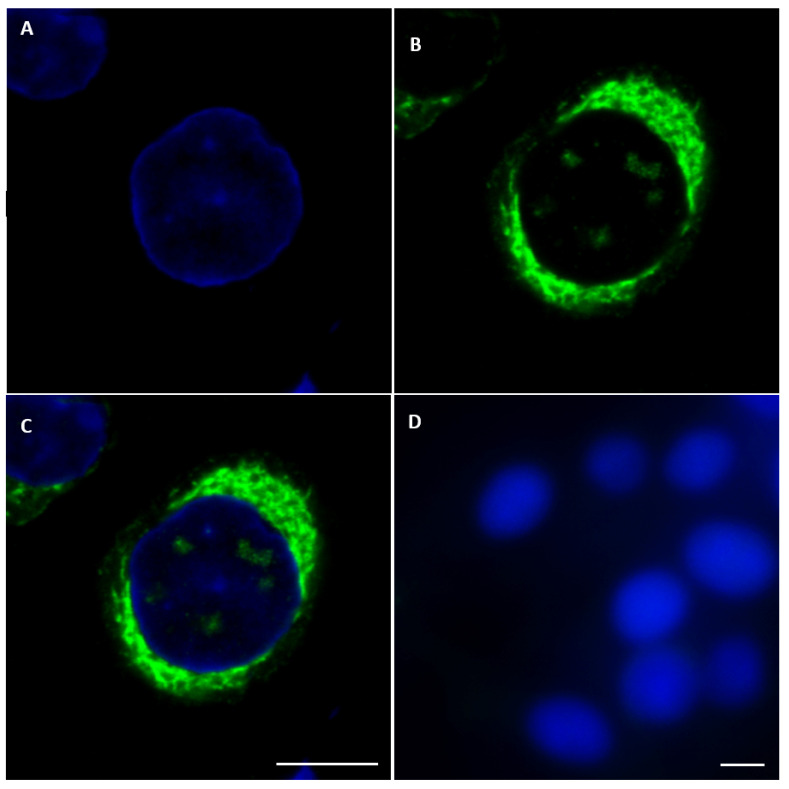
Confocal immunofluorescence microscopy analysis of NS5 expression in BTV-1-infected BSR cells. Anti-NS5 antibodies identify NS5 both in the cytoplasm and, to a lesser extent, in the nucleus. Mouse serum was adsorbed onto non-infected BSR cells to remove any potential non-specific antibodies. (**A**): nuclei coloured by DAPI; (**B**): NS5 signal identified by mouse anti-NS5 of BTV-1 and revealed by Alexa Fluor 488 (green fluorescence) conjugated anti-mouse Ig; (**C**): a merge of (**A**,**B**) panels; (**D**): non-infected BSR cells tested with anti-NS5 of BTV1 and Alexa Fluor 488 conjugated anti-mouse IgG (negative for NS5 detection). The scale bar represents 5 µm.

NS5 of BTV-1 was identified in both the cytoplasm (intense green fluorescence) and at lower levels in the nucleus of BSR cells infected with BTV-1RG_C7_, using anti-NS5 anti-sera (Figure 6 and Figure S9 and NS5 Movie 1). Similarly, BSR cells infected with a distinct BTV serotype (BTV-8RSArrrr/08) showed an NS5 signal in both the cytoplasm and, to a lesser extent, the nucleus (Figure S10).

The anti-BTV-NS5 antibodies did not detect NS5 in BSR cells infected with the NS5 deletion mutant BTV-1ΔNS5 (Figure S11A), although NS4 expression was detected using anti-NS4 antibodies (Figure S11B). This confirms infection and virus replication.

#### 2.3.2. AHSV NS5

**Figure 7:**
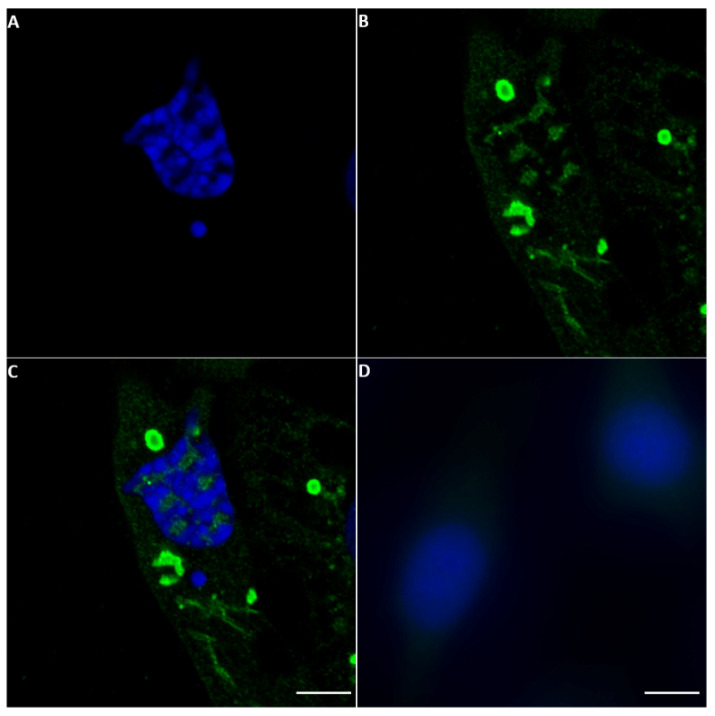
Immunofluorescence microscopy analysis of NS5 expression in AHSV-4 Morocco-infected BSR cells. Anti-NS5 antibodies from AHSV-4 identified NS5 in both the cytoplasm and nucleus of infected BSR cells by confocal microscopy. (**A**): nuclei coloured by DAPI. (**B**): NS5 is identified by anti-NS5 AHSV-4 antibodies and Alexa Fluor 488 (green fluorescence) conjugated anti-mouse IgG. (**C**): a merge of (**A**,**B**) panels and (**D**): non-infected BSR cells tested with anti-NS5 AHSV-4 antibodies and Alexa Fluor 488 conjugated anti-mouse IgG (negative for NS5 detection).

NS5 of AHSV was also identified in both the cytoplasm and the nucleus of BSR cells infected with AHSV-4 Morocco by staining with anti-AHSV-4-NS5 anti-sera (Figure 7 and Figure S12). Confocal fluorescence microscopy indicated that the distribution of AHSV NS5 in the cell cytoplasm is not even or random but appears to be very localised. The staining of ring-shaped structures is reminiscent of viral inclusion bodies (VIBs) stained with anti-NS2 antibodies seen in earlier studies [36]. Future studies will be performed using antibodies to the bacterially expressed NS2 of BTV and/or AHSV to test the possibility that NS5 interacts with the VIB.

#### 2.3.3. KEMV NS5

**Figure 8:**
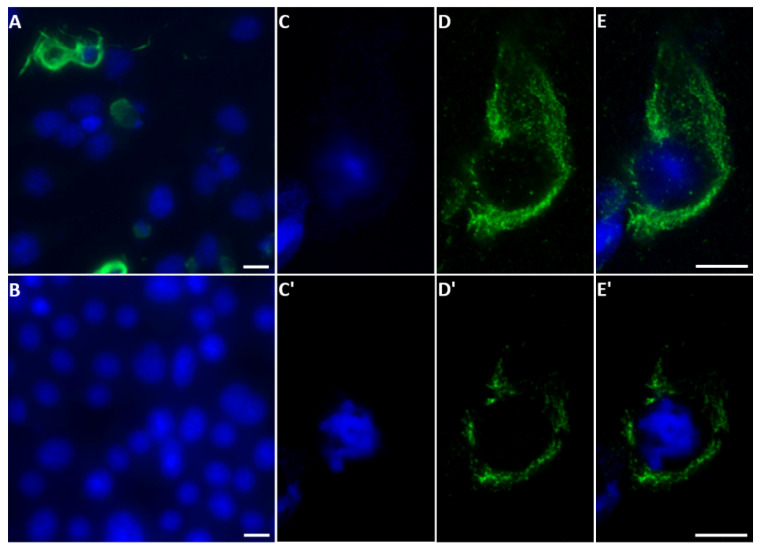
Detection of NS5 in KEMV-infected BSR cells by fluorescence microscopy: Nuclei were stained with DAPI and probed with mouse anti-KEMV NS5 antibodies and Alexa Fluor 488-conjugated anti-mouse IgG. Epifluorescence microscopy of immunofluorescence microscopy of KEMV-infected (**A**) and non-infected (**B**) BSR cells, using anti-NS5 KEMV antibodies. NS5 is mainly identified in the cytoplasm of BSR cells infected with KEMV. Confocal immunofluorescence microscopy of KEMV-infected BSR cells using anti-NS5 anti-sera (**C**–**E** and **C′**–**E′**). Two focal planes of the same cell are shown. (**C**,**C′**) show the nuclei coloured by DAPI. (**D**,**D′**) show the detection of NS5 (green fluorescence). (**E**,**E′**) show a merge of the (**C**,**C′**) and (**D**,**D′**) panels. The scale bar represents 5 µm.

With confocal immunofluorescence microscopy, anti-sera against NS5 of KEMV were used to identify NS5 of KEMV in KEMV-infected BSR cells (Figure 8A). Observation of two focal planes of the same infected BSR cells (Figure 8C–E,C′–E′) confirmed that KEMV NS5 localises principally to the cytoplasm, with a relatively uniform distribution and a lower and more dispersed punctate signal in the nucleus. This was confirmed by a z-stack confocal analysis (NS5 Movie 2).

#### 2.3.4. EHDV NS5

**Figure 9:**
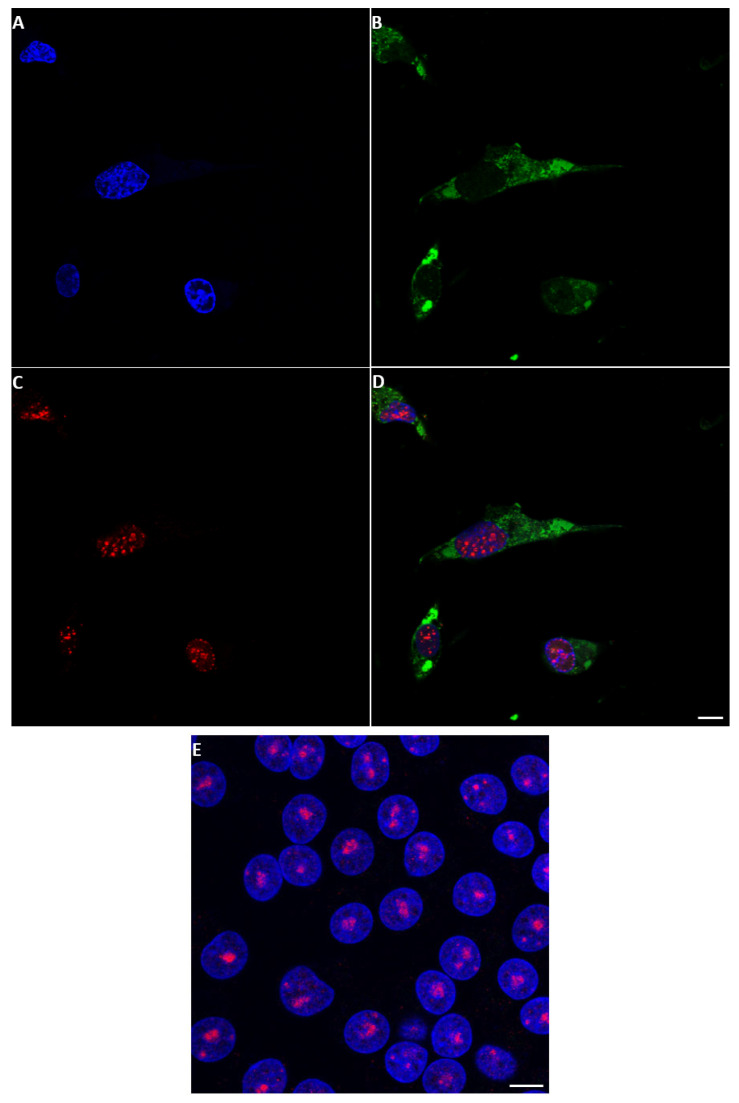
Detection of EHDV-NS5 expression by confocal immunofluorescence microscopy of infected BSR cells. Anti EHDV-NS5 anti-sera identified NS5, mainly in the cytoplasm of EHDV-infected BSR cells. (**A**): EHDV-infected BSR cells stained with DAPI. (**B**): cells probed with anti-EHDV NS5 antibodies and Alexa Fluor 488-conjugated anti-mouse IgG (green). (**C**): cells probed with anti-fibrillarin antibodies and Alexa Fluor 568 anti-rabbit IgG. (**D**): merge of (**A**–**C**). Panel (**E**) shows non-infected BSR cells stained with DAPI and probed with anti-EHDV NS5 antibodies, Alexa Fluor 488 anti-mouse IgG, anti-fibrillarin antibodies, and Alexa Fluor 568 anti-rabbit IgG. The scale bar represents 5 µm.

EHDV NS5 was also identified by confocal immunofluorescence in BSR cells infected with EHDV-7Is using anti-EHDV-7-NS5 antiserum and appears to localise mainly in the cytoplasm (Figure 9) with some punctate distribution in the nuclei. Probing infected cells with anti-fibrillarin and anti-NS5 antibodies failed to localise the punctate nuclear spots of NS5 to the nucleoli.

### 2.4. NS5 Localises to the Nucleolus during Early Stages of Orbivirus Infections

During the early stages of BTV-1 infection in BSR cells (12–14 h), NS5 was detected in the nucleus and appeared to localise to the nucleoli. However, as infection progressed (16 h post-infection), NS5 accumulated throughout the nucleus, particularly in the vicinity of nucleolar matter, and nucleolar structures appeared smaller (as compared to non-infected cells) (Figure S13).

**Figure 10:**
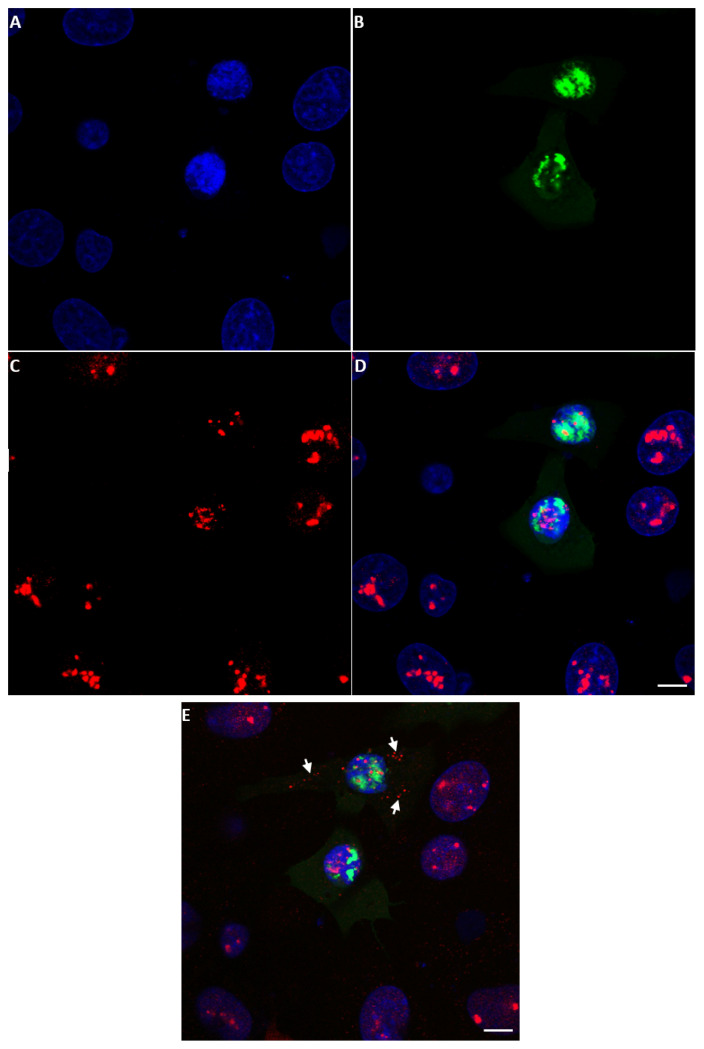
Confocal immunofluorescence microscopy of BSR cells transfected with pCI-BTV1NS5-GFP (expressing NS5-GFP of BTV-1) at 18 h post-transfection. (**A**): cells were stained with DAPI. (**B**): green fluorescence of the NS5-GFP fusion protein is visible in the nucleus. (**C**): fibrillarin in the nucleoli, and (**D**): the merging of (**A**–**C**). The green fluorescence of the fusion protein is located in the vicinity of nucleolar material (red fluorescence: anti-fibrillarin antibodies) and throughout the nucleus. A different focal plane (**E**) shows the top cell expressing the NS5-GFP fusion protein having punctate nucleolar matter (red) both in the nucleus and the cytoplasm (indicated by white arrows). The scale bar represents 5 µm.

BSR cells transfected with plasmid pCI-BTV1NS5-GFP also showed smaller nucleolar structures upon accumulation of NS5-GFP, as compared to neighbouring non-transfected cells (Figure 10). At 18 h post-transfection, the larger structure of the nucleolus appeared disorganized and smaller nucleolar structures were revealed by anti-fibrillarin antibodies both in the nucleus and the cytoplasm (Figure 10E). A z-stack confocal analysis clearly shows the presence of the smaller nucleolar material both in the nucleus and the cytoplasm (NS5 Movie 3).

### 2.5. Co-Expression of NS5 and NS4 or VP5 in BSR Cells

BSR cells were co-transfected with plasmids pCI-BTV1NS4 and pCI-BTV1NS5. The cells were then probed with mouse anti-NS5 antibodies and rabbit anti-NS4 antibodies. Cells co-expressing the two proteins are shown in supplementary Figure S14. The two proteins appear to co-localise, as shown in the merged images. The calculated value of Pearson’s coefficient using ImageJ was up to 0.86, confirming co-localisation.

BSR cells were transfected with the dual cassette expression plasmid pCI-BTV1NS5-GFP/BTV1VP5 and probed with mouse anti-VP5 anti-sera/Alexa Fluor 568-conjugated anti-mouse IgG. The NS5-GFP signal was mainly observed in the nucleus, while the VP5 signal was solely observed in the cytoplasm of transfected cells (Figure S15). Of particular interest, VP5 appears as discrete blobs and is not uniformly distributed in the cytoplasm.

### 2.6. Localisation of NS5 to Mitochondria

**Figure 11:**
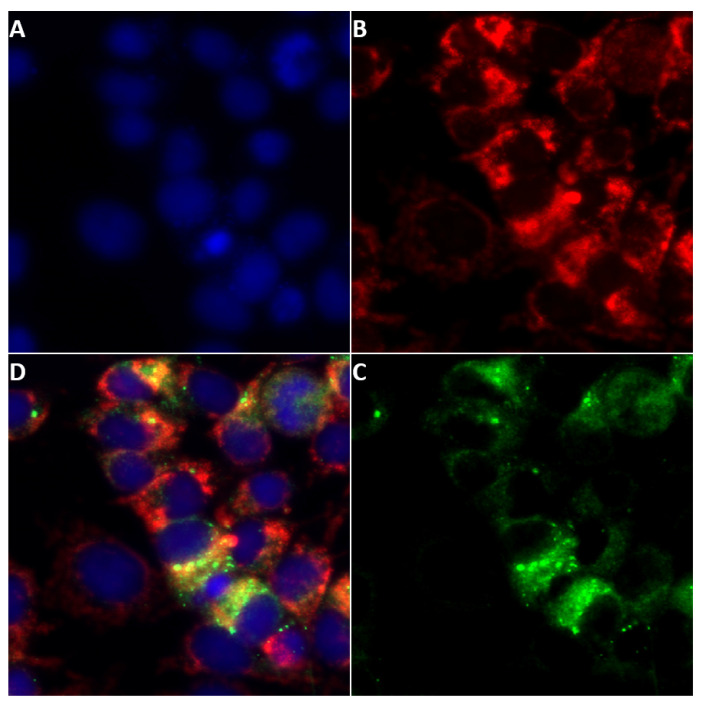

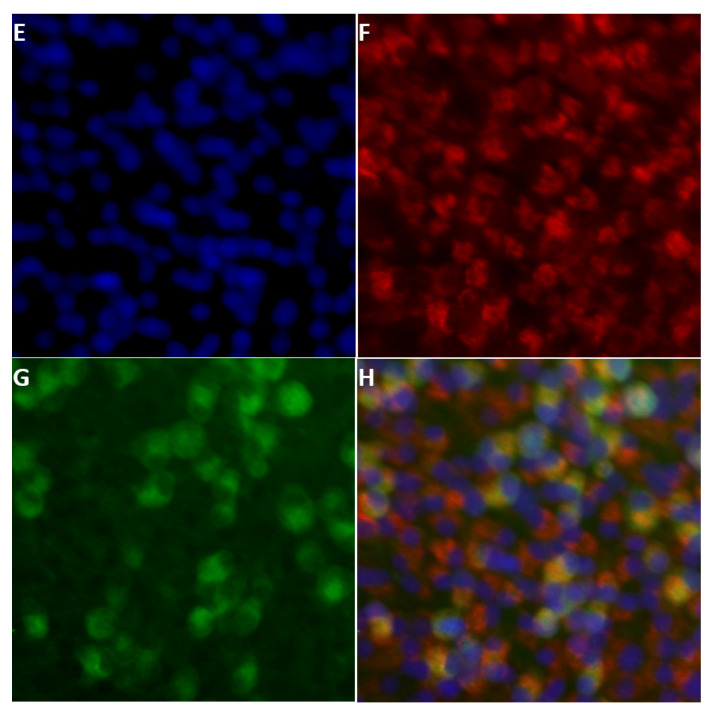
Co-localisation of NS5 with mitochondria in BSR cells infected with BTV-1 or AHSV-4. Infected cells were stained with MitoTracker^®^ Red CMXRos (red) and probed with mouse anti-NS5 anti-sera and Alexa Fluor 488 conjugated anti-mouse IgG (green fluorescence). Panels (**A**–**D**): Immunofluorescence microscopy analysis of BSR cells infected for 24 h with AHSV-4. Panels (**E**–**H**), BSR cells infected for 24 h with BTV-1. Cells were stained with DAPI (**A**,**E**), with MitoTracker^®^ Red CMXRos (red) staining the mitochondrial matrix (**B**,**F**); probed with anti-AHSV-NS5 antibodies, panel (**C**) (green), or anti-BTV-NS5 panel (**G**) (green). Panel (**D**): merge of (**A**–**C**); panel (**H**): merge of (**E**–**G**). Both (**D**) and (**H**) show co-localised NS5 and mitochondria (yellow).

Our bioinformatic analysis suggested that orbivirus NS5 proteins have the potential to localise to the mitochondrial matrix. Cells infected with BTV-1 or AHSV-4 for 24 h and stained with MitoTracker^®^ Red CMXRos and mouse anti-NS5 antibodies, were used to identify NS5 distribution. The results shown in Figure 11 indicate that NS5 does localise to the mitochondrial matrix, with a calculated Person coefficient of 0.68 and 0.65 for AHSV and BTV, respectively.

### 2.7. Pulldown of Protein-Protein Complexes in BSR Cells Expressing His-Tagged NS5

His-tagged NS5 proteins of BTV-1 or AHSV-4 were expressed in BSR cells after transfection with plasmids pCI-BTV1NS5-6xHis or pCI-AHSVNS5-6xHis. Protein complexes pulled down from the lysates of these cells using a Dynabeads^®^ His-Tag Isolation and Pulldown kit were analysed by Western blotting using anti-6xHis antibodies. This identified protein bands of ~15 kDa that were absent from non-transfected lysates, confirming the expression and pulldown of NS5-6xHis proteins (Figure S8). The AHSV NS5 appears larger than the BTV NS5, in agreement with molecular sizes calculated from their amino acid sequences.

**Figure 12:**
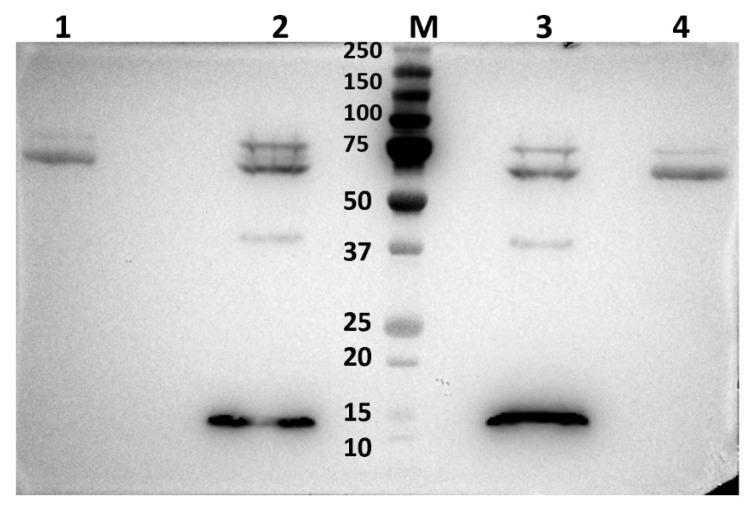
Detection of NS5-6xHis and ZBP1 by Western blotting in protein complexes pulled from BSR cell lysates expressing NS5-6xHis. Lanes 1 and 4 show Western blots of products pulled from lysates of non-transfected BSR cells. Lane 2: contains products pulled from lysates of BSR cells expressing BTV-1 NS5-6xHis (transfected with plasmid pCI-BTV1NS5-6xHis). Lane 3: products pulled from lysates of BSR cells expressing AHSV-4 NS5-6xHis (transfected with plasmid pCI-AHSVNS5-6xHis). In each case, protein complexes were pulled down using Dynabeads as described in materials and methods, which were then analysed by Western blotting using anti-his-tag antibodies and anti-ZBP1 antibodies. Protein bands that migrated in appropriate positions for NS5-6xHis (~15 KDa), and ZBP1 (~40 KDa), were detected in both lanes 2 and 3, indicating an association of these proteins in the transfected cell. Non-specific bands having sizes of about 65 and 75 kDa were also detected in non-transfected cells in lanes 1–4. These non-specific bands can be observed in Western blots of BSR lysates, both mock-transfected and transfected with pCI-BTV1NS5-6xHis (Figure S17).

Our bioinformatic analyses have identified similarities between the aa sequence of NS5 and the ZBP1 Z-alpha domain. We found that an anti-human ZBP1 antibody (Sigma, Saint-Quentin-Fallavier, France) cross-reacts with a hamster’s ZBP1, detecting two isoforms of this protein (Figure S16). The BSR lysates expressing BTV-1 or AHSV-4 NS5-6xHis pulldown products probed with the anti-ZBP1 antibody detected a band with a molecular weight of ~40 kDa (Figure 12), consistent with the smallest of the two isoforms detected by the antibody. This 40 kDa band was absent from the negative control pulldown of non-transfected BSR cell lysates (Figure 12). This finding indicates that the 40 kDa band representing hamster ZBP1 had complexed with NS5 in the transfected cells.

### 2.8. Replication of BTV-1RG_C7,_ BTV-1ΔNS5, BTV-1ΔNS4 or BTV-1ΔNS5/ΔNS4 in BSR Cells

**Figure 13:**
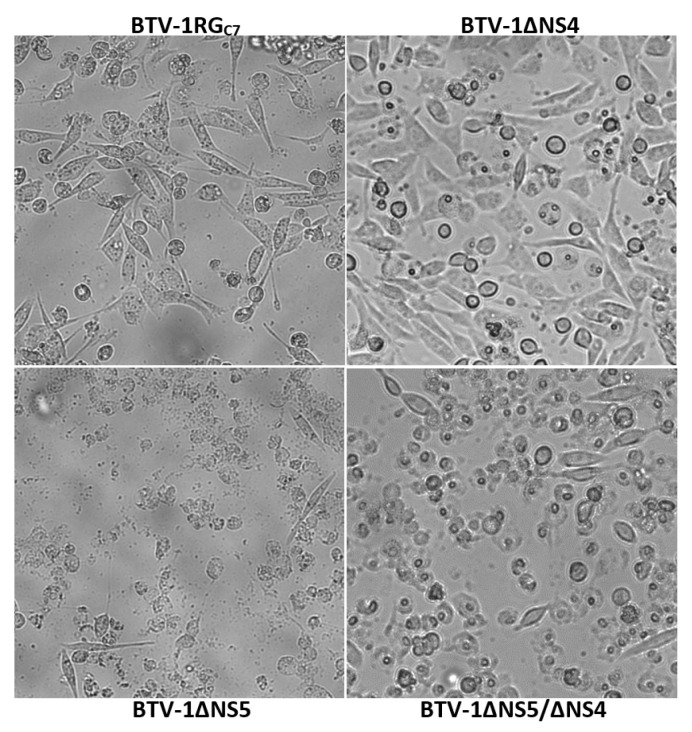
Light microscopy of BSR cell monolayers infected with BTV-1RG_C7_, BTV-1ΔNS5, BTV-1ΔNS4, or BTV-1ΔNS5/ΔNS4. BSR cell monolayers were infected with BTV-1RG_C7_ (derived by reverse genetics from the wild type BTV1-RSArrrr/01), BTV-1ΔNS5, BTV-1ΔNS4 or BTV-1ΔNS5/ΔNS4 and monitored by light microscopy. The images shown were taken 48 h post-infection. Cells infected with BTV-1RG_C7_ or BTV-1ΔNS4 are significantly less lysed than cells infected with BTV-1ΔNS5 or BTV-1ΔNS5/ΔNS4.

At 48 h post-infection with either BTV-1RG_C7_ or BTV-1ΔNS4, similar levels of cell lysis/death were observed by light microscopy in BSR cells (Figure 13). However, damage to the cells appeared significantly more severe/advanced in cultures infected with BTV-1ΔNS5 or BTV-1ΔNS5/ΔNS4.

**Figure 14:**
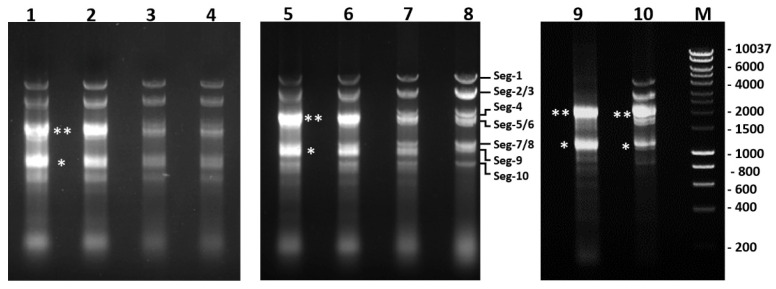
Analysis of total RNA extracted from BSR cells infected with BTV-1RG_C7_, BTV-1ΔNS4 or BTV-1ΔNS5 at 34 h post-infection. Total RNA from BSR cells infected with BTV-1RG_C7_ (lanes 1, 5 and 10), BTV-1ΔNS4 (lanes 2 and 6), BTV-1ΔNS5 (lane 3), or BTV-1ΔNS5/ΔNS4 (lane 4) analysed by agarose gel electrophoresis. Lanes 1 and 2 contained similar amounts of viral dsRNA, which were higher than those visible in lanes 3 and 4. Lane 7: total RNA extract of BTV-1ΔNS5 infected cells (five times the quantity shown in lane 3). Lane 8: dsRNA of BTV-1RG_C7_, in which 28S (**) and 18S (*) rRNA bands had been removed by LiCl purification. Lanes 9 and 10: total RNA extracts of non-infected or BTV-1RG_C7_ infected BSR cells, respectively. Lanes 3, 4, and 7 show little rRNA as compared to lanes 1, 2, 5, 6, 9, and 10. Lane M: size marker labelled in bp. The 28S and 18S rRNA bands are indicated by ** and * respectively.

Analysis by agarose gel electrophoresis (AGE) of total RNA showed similar quantities of the viral dsRNA segments (virus genome) in extracts from BSR cultures infected with BTV-1RG_C7_ and BTV-1ΔNS4. This contrasts with a lower level of genomic dsRNA in cultures infected with BTV-1ΔNS5 or BTV-1ΔNS5/ΔNS4 (Figure 14). The levels of ribosomal RNAs detected in extracts from infected BTV-1ΔNS5 or BTV-1ΔNS5/ΔNS4 cells were also significantly reduced (Figure 14).

Real-time RT-PCR assays targeting BTV genome segment 10 showed similar mean Ct values at 34 h post-infection of BSR cells with either BTV-1RG_C7_ or BTV-1ΔNS4 (of 6.39 and 6.78, respectively). However, ~4.5 Ct higher values were observed for BSR cells infected with BTV-1ΔNS5 or BTV-1ΔNS5/ΔNS4 (mean Ct values of 10.98 and 10.95, respectively). This is in line with the different amounts of viral dsRNA generated in BSR cells, as observed by AGE (Figure 14), indicating a reduction in the production of progeny virus particles in the absence of NS5. In contrast, similar mean Ct values (~10 Ct) were detected in KC cell cultures at 6 days post-infection with BTV-1RG_C7_, BTV-1ΔNS5, BTV-1ΔNS4, or BTV-1ΔNS5/ΔNS4.

AGE analysis of the total RNA extracted from the BSR cell cultures infected with BTV-1RG_C7_ or BTV-1ΔNS4, showed large quantities of both 28S and 18S rRNAs. In contrast, little ribosomal RNA was detected in extracts from BTV-1ΔNS5 infected BSR cell cultures, even at 5 times higher loading (Figure 14). This indicates that NS5 protects the integrity of the ribosomal RNAs. The levels of ribosomal RNAs detected in RNA extracts from KC cells infected with BTV-1RG, BTV-1ΔNS5, BTV-1ΔNS4, or BTV-1ΔNS5/ΔNS4 were all similar. It is noteworthy that BSR cells are lytically infected while KC cells are persistently infected and would therefore need to maintain ribosomal activity despite infection to maintain cell viability.

### 2.9. Replication of Recombinant Vaccinia Expressing NS5 in A549 Cells

**Figure 15:**
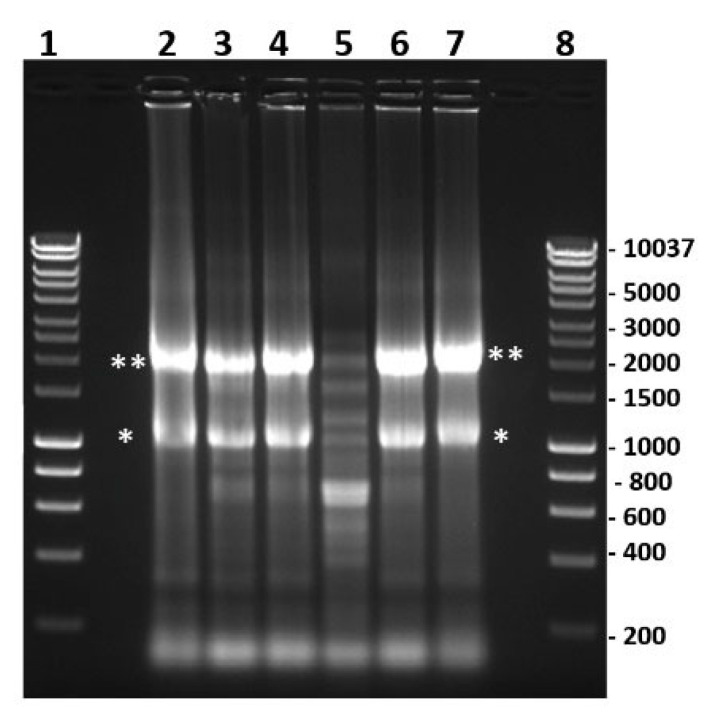
Ribosomal RNA profile of A549 cells infected with wild-type and recombinant vaccinia viruses at 24 h post-infection. Lanes 1 and 8: DNA size marker. Lanes 2 and 7: non-infected A549 cells. Lane 3: wild-type VVC. Lane 4: VV-VP1080-E3L. Lane 5: the ΔE3L virus VV-VP1080. Lane 6: VV-VP1080-NS5BTV1. The positions of 28S rRNA and 18S rRNA are indicated with double and single asterisks, respectively. Lane 5, where cells were infected with the ΔE3L virus VV-VP1080, shows extensive degradation of both 28S and 18S rRNA, although adding back the E3L or NS5 gene into VV-VP1080 (VV-VP1080-E3L or VV-VP1080-NS5BTV1) protected rRNA from degradation.

RNA preparations extracted from non-infected human lung carcinoma A549 cells or cells infected with wild-type vaccinia virus Copenhagen (VVC), VV-VP1080 (a VVC E3L deletion mutant), or recombinant vaccinia viruses VP1080-NS5BTV-1 (generated by homologous recombination to express BTV NS5) or VV-VP1080-E3L (generated by homologous recombination to express E3L), were analysed by 1% agarose gel electrophoresis (Figure 15). Ribosomal RNA integrity did not appear to be compromised in cells infected with wild-type VVC, VV-VP1080-E3L, or VP1080-NS5BTV-1, while cells infected with the ΔE3L virus VV-VP1080 showed extensive degradation of ribosomal RNA, indicating that E3L and BTV-NS5 both protect the integrity of cellular ribosomal RNA.

### 2.10. Assessment of Protein Synthesis in BSR Cells Infected with BTV-1RG_C7_ or BTV-1ΔNS5

**Figure 16:**
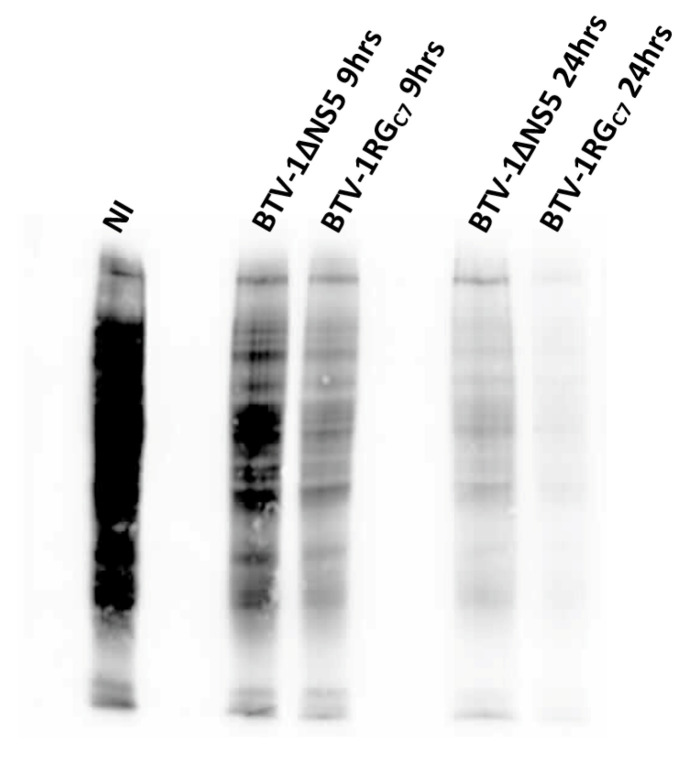
Pulse and chase puromycin labelling of cells infected with BTV-1RG_C7_ or BTV-1ΔNS5. BSR cells infected with BTV-1RG_C7_ or BTV-1ΔNS5 were pulse-labelled with puromycin for 15 min at 9 or 24 h post-infection (as indicated), then harvested 3 h later. Western blot analysis was performed using a monoclonal anti-puromycin antibody. NI indicates analysis of non-infected cells.

Puromycin labelling is a reliable alternative to radiolabeling used to assess global protein synthesis in cells [37,38]. At 9 h post-infection, BSR cells infected with BTV-1RG_C7_ or BTV-1ΔNS5 showed significant inhibition of global protein synthesis compared to non-infected cells, as previously reported for BTV-infected mammalian cells [39,40]. By 24 h post-infection, overall protein synthesis was further reduced, with a greater reduction in the BTV-1RG_C7_ infected cells compared to the BTV-1ΔNS5 infected cells (Figure 16). This difference in overall levels of protein synthesis may in part be due to lower levels of replication and the lower titres of BTV-1ΔNS5 generated. However, it does not appear to correlate with the degradation of ribosomal RNAs observed at 34 h pi (see Figure 14) or the higher percentage of lysed cells observed at 48 h pi in BTV-1ΔNS5-infected cells (Figure 13), both of which would be expected to reduce levels of cellular protein synthesis. This suggests that NS5 plays a significant role in a different mechanism involved in the overall shut-off of host cell protein synthesis seen in BTV-infected mammalian cells, possibly by acting as a repressor of cellular RNA transcription. Together with the increased cell viability and reduction in cell lysis (Figure 13), NS5 appears likely to promote overall viral protein synthesis and replication when it is expressed in BTV-infected cells.

### 2.11. Nuclease Treatment of Bacterially Expressed and Purified NS5

**Figure 17:**
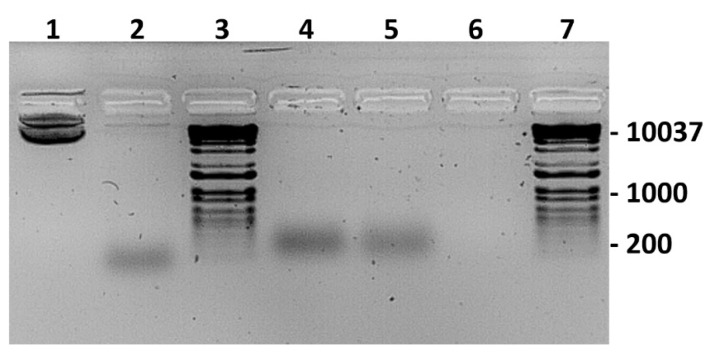
Agarose gel electrophoresis of GST-fused NS5 of BTV-8. Lane 1: pACT-5C plasmid (250 ng); lane 2: pACT-5C plasmid (250 ng) treated with Turbo DNAse (0.2 units, 1× reaction buffer, 1 h at 37 °C) showing degradation products; lanes 3 and 7: DNA size marker; lane 4: GST-fused NS5 of BTV-8 (denatured by boiling for 10 min); lane 5: GST-fused NS5 of BTV-8 (denatured by boiling for 10 min) treated with Turbo DNAse (0.2 units, 1× reaction buffer, 1 h at 37 °C); and lane 6: GST-fused NS5 of BTV-8 (denatured by boiling for 10 min) treated with RNAse A (0.4 µg in 1× reaction buffer, 1 h at 37 °C). The products were run in a 1.5% agarose gel in 0.5× TBE buffer.

Our bioinformatic analyses suggest that NS5 shares similarities with known nucleic acid binding proteins (Figure 1 and Figure 4). Analysis of the bacterially expressed GST-NS5 proteins by agarose gel electrophoresis in the presence of ethidium bromide showed that nucleic acids were bound to the purified protein (Figure 17). Multiple treatments of bacterially expressed NS5 proteins with 1M NaCl before elution from glutathione sepharose during purification did not release the bound bacterial RNAs, suggesting that it binds to NS5 with high affinity.

RNAse A is a phosphodiesterase that cleaves phosphodiester bonds in RNA, releasing nucleotides. DNase I cleaves DNA in a sequence-independent manner, releasing 5′-phosphorylated di-, tri-, and oligonucleotides [41]. Treatment of purified recombinant NS5 with nucleases demonstrated that RNAse A (Roche) but not RNAse-free DNAse I (Turbo DNAse, Thermo Fisher, les Ulis, France) degraded the bound nucleic acids, identifying them as RNA (Figure 17). However, the nucleotide sequence specificity of this interaction, if any, will require further characterisation.

### 2.12. Electromobility Shift Assays (EMSA)

**Figure 18:**
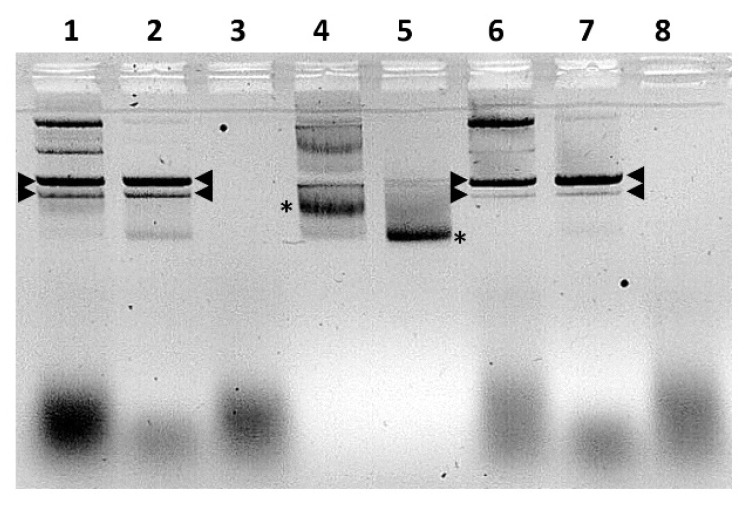
Electrophoretic mobility shift assays after interaction with GST-fused NS5 of KEMV or BTV-8. Protein-DNA binding reactions were performed in 0.5× TBE buffer containing 100 mM NaCl and 5 mM MgCl_2_ for 30 min at room temperature. The reaction products were analysed by electrophoresis in 1% agarose gel containing TBE 0.5× and 100 mM NaCl. Lane 1: pCIneo plasmid (100 ng) plus 100 ng of KEMV GST-fused NS5; lane 2: pCIneo-24CG plasmid (100 ng) plus 100 ng of KEMV GST-fused NS5; lane 3: GST-Fused NS5 of KEMV; lane 4: pCIneo plasmid (100 ng); lane 5: pCIneo-24CG plasmid (100 ng); lane 6: pCIneo plasmid (100 ng) plus 100 ng of BTV-8 GST-fused NS5; lane 7: pCIneo-24CG plasmid (100 ng) plus 100 ng of BTV-8 GST-fused NS5; lane 8: GST-fused NS5 of BTV-8. Nucleic acids were visualised after electrophoresis by UV transillumination in the presence of ethidium bromide. Supercoiled forms of the pCIneo or pCIneo-24CG are indicated by asterisks. Plasmid-NS5 complexes are indicated by arrow heads.

Purified expressed NS5 proteins from BTV or KEMV were incubated with the plasmid pCIneo or a version of pCIneo containing a DNA fragment consisting of 24 CG dinucleotide repetitions that assume a Z-DNA form (designated pCIneo-24CG [42]). Interactions with either NS5 protein altered the migration of both pCIneo or pCIneo-24CG DNA (Figure 18), as analysed by electrophoretic mobility shift assays in agarose gels (Figure 18).

Nucleic acid binding proteins may require divalent cations for efficient binding of nucleic acids [43]. Therefore, we assessed the effect of magnesium, calcium, and zinc ions (as chloride solutions) on the binding of pCIneo-24CG to NS5 of BTV, by EMSA. The addition of 5 mM MgCl_2_ increased the bound form of the plasmids, with a decreased intensity of the free form (Figure S18, lane 2). The addition of CaCl_2_ or ZnCl_2_ did not improve binding to the protein (Figure S18), and the addition of ATP (150 µM) or DTT (1 mM) had no effect on binding. Multiple preparations of AHSV NS5 also caused electrophoretic mobility shifts of the pCIneo-24CG plasmid, indicating a high affinity for nucleic acids (Figure S19). Further studies indicated that the GST-fused NS5 proteins of BTV or KEMV do not bind linear PCR products (Figures S20 and S21) or BTV genomic dsRNA (Figure S22). A lower concentration of BTV-NS5 (~5 ng) interacted efficiently with supercoiled plasmids and still caused an electrophoretic mobility shift, particularly in the supercoiled forms (Figure S23).

The EMSA results suggest that NS5 of BTV, AHSV, or KEMV preferentially binds supercoiled forms of plasmid DNA. Therefore, it appears likely that NS5 can also interact with condensed/supercoiled forms of cellular DNA, in agreement with the confocal immunofluorescence microscopy data. This shows the accumulation of these proteins in the nucleus during infections or when ectopically expressed from a transfected plasmid.

### 2.13. Transfection of BSR Cells with pCI-BTV1NS5-6xHis or pCI-AHSVNS5-6xHis and Infection with BTV-1RG_C7_ or BTV-1ΔNS5

BSR cells infected with BTV-1ΔNS5 were also transfected with plasmids expressing NS5-6xHis of BTV-1 or AHSV-4, and their lysates were incubated with Dynabeads^®^ from the His-Tag Isolation and Pulldown kit. After pulldown, RNA was extracted from the beads and tested for BTV-RNA using RT-PCR assays targeting genome segments 1, 3 and 7. A significant decrease in Ct values (between 3.5 and 8.4 Ct reduction) was observed between the non-transfected but BTV-1ΔNS5 infected control cells and the BTV-1ΔNS5 infected cells that were also transfected with plasmids expressing the NS5-6xHis of BTV-1 or AHSV-4 (Table 2a), indicating that BTV RNA had bound to the NS5-6xHis. The NS5-6xHis did not increase the overall efficiency of viral RNA synthesis, as confirmed by real-time RT-PCR, performed using total RNA extracts of infected BTV-1ΔNS5-infected BSR cells. BSR cells were also transfected with plasmids expressing NS5-6xHis of BTV-1 or AHSV-4, followed by infection with BTV-1RG_C7_. The observed Ct values indicate that there are no differences between transfected and non-transfected cells, likely due to the abundant expression of native, non-tagged NS5 by BTV-1RG_C7_ during infection (Table 2b).

Therefore, the plasmid-expressed NS5-6xhis proteins of BTV-1 or AHSV-4 appear to bind significant amounts of BTV-1 mRNA in cells infected with BTV-1ΔNS5. The similar efficiencies indicate that mRNA binding by NS5 is not virus species-specific.

## 3. Discussion

An overlapping ORF in genome segment 10 (S10-ORF2) of BTV has previously been predicted by bioinformatic analysis using the programme FRESCo [26]. A study by Stewart et al. [27] provided an initial characterisation of the potential translation product’s properties but did not directly confirm the existence of the protein in BTV-infected cells. Using a reporter gene assay, it was proposed that the product encoded by S10-ORF2 inhibited gene expression of transfected plasmids but not RNA translation. It was also suggested that an S10-ORF2 deletion mutant of BTV-8NET2006/04 does not influence the replication kinetics of the virus in mammalian or insect cells and does not alter the pathogenicity in IFNAR^(−/−)^ mice [27].

We present data confirming the expression and identification of the S10-ORF2 translation product (which we designate as non-structural protein 5 or NS5) in cells infected with BTV, EHDV, AHSV, or KEMV, implying a conserved role in orbivirus replication. Our bioinformatics analyses of the NS5 sequences show that it is similar to proteins that bind to nucleic acids. In particular, the predicted NS5 secondary structure is similar to the Z-alpha domains of the Z-alpha protein family, which represents a large number of proteins, including the cellular ZBP1 (also known as DAI or DLM-1), the adenosine deaminase ADAR1 (RNA editing enzyme), and viral proteins that include the E3L protein of poxviruses [44,45]. The longer forms of NS5, particularly AHSV NS5, have a similar size and share conserved motifs with the full-length Z-alpha domain of ZBP1 or E3L.

ZBP1 is a sensor of the Z-form of nucleic acids (DNA or RNA) and is constitutively expressed in the cytoplasm of tissues such as the lungs, spleen, liver, small intestines, and lymphatic tissues [46,47]. In virus-infected cells (orthomyxoviruses, herpesviruses, flaviviruses), ZBP1 binds viral Z-RNA and induces an interferon response or triggers pathways of cell death, including apoptosis, pyroptosis, and necroptosis [30]. ZBP1 associates with bodies that are involved in the regulation of mRNA turnover, including cytoplasmic stress granules [48,49]. In contrast, NS5 appears to help maintain cellular integrity and the levels of ribosomal RNA, which are likely to help promote viral protein synthesis and replication.

Bioinformatic analyses predict that NS5 of BTV contains multiple monopartite NLSs with high scores, while NS5 of EHDV and AHSV contain only one predicted NLS with intermediate scores. In BTV- or AHSV-infected or plasmid transfected BSR cells, confocal immunofluorescence microscopy identified NS5 in the nucleoli and throughout the nucleus, as well as in the cytoplasm, while in EHDV- or KEMV-infected cells, NS5 was primarily detected in the cytoplasm. Even though no monopartite NLS signals were identified in NS5 of KEMV, bipartite NLSs were predicted in NS5 of both BTV and KEMV (BTV with a high score and KEMV with a threshold score).

The nucleolus is the site of ribosome biogenesis. It is also involved in apoptosis, stress response, and cell cycle regulation [50]. During the early stages of infection of BSR cells with BTV, NS5 localises to the nucleoli, further spreading across the nucleus and into the cytoplasm. Ectopically expressed GFP-fused BTV NS5 also localises to the nucleoli of BSR cells, confirming predictions for ovine cells by Stewart et al. [27]. However, as NS5 accumulates in the nuclei of BSR cells, smaller nucleolar structures (revealed by probing with anti-fibrillarin antibodies) replace the larger nucleoli and are visible both in the nuclei and the cytoplasm.

Previous radiolabeling studies have demonstrated that infection of cells with dsRNA viruses induces shut-off of host-cell protein synthesis, resulting in radiolabelled amino acids being incorporated mainly into nascent viral proteins [39,51]. The mRNAs of viruses belonging to the families *Spinareoviridae* and *Sedoreoviridae* are not polyadenylated, and the circularization of the mRNA bound to translation initiation complexes does not require polyA-binding protein (PABP). Viral non-structural proteins such as rotavirus NSP3 [52] or orbivirus NS1 [19] bind conserved sequences within the 3′-non-coding regions of the viral mRNAs, promoting translation, in a manner similar to PABP with the polyadenylated cellular mRNA [52,53]. This differentiates viral and host cell RNAs and may be involved in specific inhibition of the host cell rather than viral protein synthesis [19,52].

Adenovirus protein V associates with the nucleoli, causing redistribution of nucleolar proteins such as nucleoline in infected cells [54], inhibiting the maturation of rRNA and protein synthesis. The wild-type BTV-1 virus causes a greater inhibition of host-cell protein synthesis in infected BSR cells than BTV-1ΔNS5. The ability of BTV NS5 to disorganize the nucleoli of BSR cells is likely to affect ribosome biogenesis, preventing the formation of novel ribosomes, and appears to correlate with reduced host cell protein synthesis. However, the ability of NS5 to also reduce the degradation of existing rRNA may allow the viral mRNAs to be preferentially translated by existing ribosomes. The possibility that NS5 downregulates the RNAse L pathway, which causes the degradation of ribosomal RNA, requires further investigation. These activities of NS5 are likely to support the role of NS1 as the primary positive regulator of viral mRNA translation in BTV-infected cells [19].

Electrophoretic mobility shift assays confirmed that NS5 interacts with nucleic acids, suggesting that its presence within the nucleus, outside the nucleolar structure, may be explained by interactions with DNA or even cellular ssRNAs. During its expression in bacteria, NS5 binds ssRNA, and although it did not interact with dsRNA in EMSA, it did induce a mobility shift of circular DNA, particularly its supercoiled form.

Eukaryotic DNA is known to be supercoiled and highly condensed [55,56]. The NS5 aa sequence has predicted similarities to cellular transcription factors. The co-transfection of plasmids expressing both NS5 and NS4 or VP5 did not prevent the expression of NS4 or VP5. However, a previous study by Stewart et al. [27] using a luciferase reporter assay, reported that NS5 inhibits transcription from various promoters (including CMV, SV40, or interferon α/β) in a dose-dependent manner. Therefore, by binding DNA and acting in a similar manner to DNA transcription regulators, NS5 could contribute to the inhibition of cellular transcription and therefore the shut-off of host-cell protein synthesis. The RNA-binding properties of NS5 could also sequester cellular mRNA in the nucleus, preventing their translation and contributing to host-cell protein synthesis shut-off.

Protein complexes “pulled down” from lysates of transfected but non-infected BSR cells expressing NS5-6xHis of BTV or AHSV contained hamster ZBP1, suggesting that NS5 interacts with ZBP1. The transfected BSR cells expressing NS5x6His of BTV or AHSV were subsequently also infected with BTV-1RG_C7_, and RNAs extracted from the pulldown complexes were subjected to real-time RT-PCR assays for viral RNAs. However, the complexes pulled from BSR cell lysates expressing NS5x6His of BTV or AHSV and super-infected with BTV-1ΔNS5, showed up to 8.0 Ct lower values for viral RNAs (equivalent to ~250 folds more RNA). This difference strongly suggests that the NS5-6xHis of BTV or AHSV binds viral ssRNA in cells infected with BTV-1ΔNS5. In cells infected with BTV-1RG_C7_, the plasmid-expressed NS5-6xHis appears to compete poorly with the authentic virally expressed NS5 for ssRNA binding, perhaps because the latter is far more abundant in the cytoplasm of infected cells. Binding viral mRNA may be important to avoid recognition by cellular sensors such as ZBP1 and could have other roles in the processing of the viral mRNAs.

ZBP1 is primarily cytoplasmic with a finely punctate distribution and appears to form nuclear foci in interferon-treated cells [48]. One isoform of ZBP1 mRNA generated by alternate splicing, which lacks the first Z-alpha domain, was found to localise to large cytoplasmic granules [48,49]. Previously published studies with mouse cytomegalovirus (MCMV) indicated that recognition of viral nucleic acids by ZBP1 in infected cells is essential to induce necroptosis. It was also shown that expression of native ZBP1 but not that with mutated Z-alpha domains helps cells defend against MCMV infection [57]. Vaccinia virus E3LΔ1-83 (Δaa 1-83: constituting the E3L Z-alpha domain) is sensed by ZBP1 to activate necroptosis [58]. It is noteworthy that the E3L protein product localises to both the nucleus and cytoplasm, inhibiting the cellular innate immune response, as well as the ZBP1 (Z-DNA binding protein 1) pathway [58,59,60,61].

Influenza viruses replicate in the nucleus of infected cells, producing Z-RNA, which is detected by ZBP1, leading to necroptosis. In influenza A virus (IAV) infected cells, ZBP1 rapidly accumulates in the nucleus. A deletion mutant of ZBP1 that lacks the Z-alpha domains fails to bind RNA and does not translocate into the nucleus. Non-structural protein 1 (NS1) of IAV interferes with the assembly of the NLRP3 (NOD-like receptor family pyrin domain containing 3) inflammasome induced by ZBP1, which was activated by Z-RNA binding, hence inhibiting necroptosis [30]. Other RNA viruses such as West Nile and Zika virus are sensed by ZBP1 in infected cells, inducing a RIPK1/RIPK3 dependent signalling pathway to block replication [30].

Therefore, it is reasonable to hypothesise that NS5 and ZBP1 either (i) directly interact with each other, (ii) indirectly interact by both binding viral ssRNA, or (iii) indirectly interact by both binding to a protein or protein-RNA complex. The ultimate outcome of this interaction is to modulate the ZBP1 signalling pathway.

The early onset of cell death in BSR cells infected with BTV-1ΔNS5, as compared to cells infected with BTV-1RG_C7_, suggests that although NS5 is non-essential for virus replication, it promotes the survival of infected BSR cells and maintains ribosomal function, therefore promoting progeny virus production.

Mitochondria are known to act as an interferon-signalling platform via the MAVS-signalling pathway, and some viral proteins can modulate this mechanism [62,63]. NS1 of IAV appears to block the interaction between RIG-I and MAVS, inhibiting RIG-I-mediated induction of interferon-β [64,65]. VP3 of BTV interacts with MAVS and IRF3-kinase IKKε and is believed to modulate the interferon antiviral response [66]. Other mitochondria-associated viral proteins encoded by DNA or RNA viruses can also play anti- or pro-apoptotic roles in infected cells [64]. NS5 of the orbiviruses was predicted to localise to the mitochondrial matrix by bioinformatic analyses and this was confirmed by Mitotracker^®^ Red staining of BSR cells infected with BTV or AHSV. In rotavirus A (RVA), genome segment 11 encodes NSP5 and NSP6 from two overlapping ORFs. NSP6 appears to localise to mitochondria [35] in infected cells, and an NSP6 deletion mutant produced a ten-fold reduction in virus titre compared to wild-type virus [34]. These findings suggest that NS5 of the orbiviruses and NSP6 of RVA may play similar functions in virus-infected cells. The rapid onset of cell death in BSR cells infected with BTV-1ΔNS5 suggests that NS5 plays a role in modulating the apoptotic process, supporting progeny virus production.

Studies are underway to further uncover the biological role(s) played by NS5 of orbiviruses in infected cells and the molecular mechanisms involved.

## 4. Materials and Methods

### 4.1. Ethics Statement

Animal experimentation protocols were approved by the Ethics Committee for animal experimentation at Anses-EnvA-UPEC (Project Licence Number: 19-028).

### 4.2. Cell Cultures and Viruses

DF1 chicken embryo fibroblasts (ATCC), baby hamster kidney BSR cells (a clone of BHK-21 cells), and A549 human lung carcinoma cells (ATCC) were grown at 37 °C in Dulbecco’s modified Eagle medium (DMEM), supplemented with 10% foetal bovine serum, 100 IU of penicillin/100 µg of streptomycin per mL, under 5% CO_2_. *Culicoides sonorensis* KC cells were grown at 28 °C in Schneider’s insect medium supplemented as above.

BTV-1RG_C7_ is a clone of BTV serotype 1, previously derived by reverse genetics based on the genome sequence of the BTV-1 reference strain [67]. BTV-8RSArrrr/08 was obtained from the orbivirus reference collection at the Pirbright Institute, UK. EHDV serotype 7 strain ISR2006 (EHDV-7) or AHSV serotype 4 Morocco (AHSV-4) were kindly provided by the orbivirus reference laboratory at Anses, France. Kemerovo virus isolate EgAn 1169-61 was kindly provided by Prof. Robert Tesh, University of Texas, Galveston, USA.

Monolayers of BSR cells (85% confluence) were infected with BTV-1RG_C7_, BTV-8RSArrrr/08, Kemerovo virus, EHDV-7, or AHSV-4 at a multiplicity of infection (MOI) of 0.05 pfu/cell, then incubated at 37 °C for 96 h. Infected cell suspensions were pelleted by centrifugation at 2000× *g* for 10 min. KC cells were infected at an MOI of 0.05 pfu/cell and then incubated at 28 °C for 7 days. Infected BSR or KC cell pellets were frozen at −80 °C until needed. RNA was extracted from cell pellets using the TRIzol reagent (Thermo Fisher) as described earlier [68].

DF1 cells were used to propagate the wild-type vaccinia virus Copenhagen (VVC) and an E3L defective VVC (designated VP1080), kindly provided by Prof. Bertram Jacobs (School of Life Sciences, Arizona State University).

### 4.3. Bioinformatic Analyses

Sequence relatedness of NS5 proteins was assessed based on data from public databases using the NCBI’s BLAST (http://blast.ncbi.nlm.nih.gov/Blast.cgi, accessed on 4 January 2023) and the Pfam software (https://www.ebi.ac.uk/interpro/search/sequence/, accessed on 4 January 2023). Amino acid alignments of NS5 from different orbiviruses were generated using the Clustal X programme [69]. Secondary structure predictions were analysed using the PredictProtein server (http://www.predictprotein.org, accessed on 22 January 2019) [70] and Jalview/Jpred [71]. Predictions of the protein fold were performed using the PHYRE2 protein fold recognition server [72] (http://www.sbg.bio.ic.ac.uk/~phyre2/html/page.cgi?id=index, accessed on 9 September 2022) or RaptorX [73] (http://raptorx.uchicago.edu, accessed on 1 November 2021). The presence of nuclear localisation signals in the amino acid sequences of NS5 from BTV, EHDV, AHSV, or KEMV and their likelihood of functionality were assessed using the programme cNLS Mapper [29] (https://nls-mapper.iab.keio.ac.jp/cgi-bin/NLS_Mapper_form.cgi, accessed on 22 January 2019), which can predict both monopartite and bipartite NLSs. The presence of nuclear localisation signals (NLS) in proteins that exclusively localise to the nucleus score > 8; proteins partially localising to the nucleus score between 6–8; proteins that theoretically localise to both the nucleus and the cytoplasm score between 3 and 5; and a score below 2 is attributed to exclusively cytoplasmic proteins.

Prediction of nucleolar localisation was performed using the nucleolar localisation signal detector NOD (http://www.compbio.dundee.ac.uk/www-nod/, accessed on 22 January 2019). The iMLP programme was used to analyse the potential targeting of proteins to the mitochondrial matrix [74] (http://imlp.bio.uni-kl.de/, accessed on 5 July 2022). Certain proteins contain internal matrix targeting signal-like sequences (iMTS-Ls), which predict their potential translocation into the mitochondrial matrix. iMLP is based on the long short-term memory (LSTM) recurrent neural network architecture. These architectures are specially designed for feature detection in sequences and are therefore well suited for the recognition of iMTS-Ls.

Co-localisation finder or JACop plugins, implemented within the FIJI programme [75], were used to quantify potential co-localisations of proteins. Pearson’s correlation coefficient, inferred using these plugins, expresses the intensity of correlation between co-localising objects in each component of a dual-colour image. Values close to 1 signify perfect correlation and co-localisation while values closer to 0 signify no correlation and hence no co-localisation.

### 4.4. Preparation of cDNAS from Orbivirus dsRNA and Cloning of the ORFs of NS4, NS5 and/or VP5 in Expression Plasmids

The dsRNAs of BTV-1, BTV-8, KEMV, EHDV-7, or AHSV-4 were all converted into cDNAs using a single primer amplification technique as previously described [68,76].

The NS5 ORF in Seg-10 of BTV-1 was PCR amplified using primers tailed with specific restriction enzyme sites to help to clone into bacterial or mammalian expression vectors (Table S1). The plasmids pGEX-4T-2 and pCI-neo, as well as NS5 ORF amplicons, were double-digested with EcoRI and NotI. Digested products were gel purified using Geneclean^®^ Kit (MP Biomedicals, Santa Ana, CA, USA). Vectors and digested PCR products were combined and ligated overnight (O/N) at 16 °C using T4 DNA ligase (Roche, Basel, Switzerland) to generate pGEX-BTV1NS5, pGEX-BTV8NS5, pGEX-KEMVNS5, pGEX-EHDV7NS5, pGEX-AHSV4NS5, pCI-BTV1NS5, pCI-BTV8NS5, pCI-BTV1NS5-6xHis, or pCI-AHSV4NS5-6xHis. The mammalian expression plasmid pCI-BTVNS4 (for expression of BTV-8 NS4) was previously described [14].

A plasmid was constructed using the “Golden Gate” cloning methodology [77] with the restriction enzyme SapI for the expression of a fusion protein containing eGFP fused to the carboxy terminal end of NS5. For that reason, plasmid pCI-NS5BTV1 was PCR amplified with primers pCI_NS5-fusion_For and pCI_NS5-fusion_Rev (Table S1). The eGFP was amplified with primers eGFP-TAG_SapI and GS3G_eGFP-ATG_SapI. The resulting plasmid is designated pCI-BTV1NS5-GFP.

The plasmid pCI-BTV1NS5-GFP/VP5 was constructed for dual expression of NS5-GFP of BTV-1 (driven by a CMV promoter) and VP5 of BTV-1 (driven by an SV40 promoter). For that purpose, the neomycin resistance gene was deleted from the pCI-BTV1NS5-GFP by PCR amplification using primers pCI-insSV40-ORF_Rev and pCI-insSV40-ORF_For, which contain SapI sites (Table S1). The ORF of Seg-6 (encoding VP5) was also PCR amplified using primers BTV1VP5-ATG_SapI and BTV1VP5-TAG_SapI containing SapI sites (Table S1). Both PCR products were ligated using the Golden Gate methodology and XL1-Blue bacteria (Agilent, Santa Clara, CA, USA) were transformed, with the recovered clones grown in LB broth containing 100 µg/mL ampicillin. Plasmids were subsequently purified using the QIAprep Spin miniprep kit (Qiagen, Hilden, Germany) and were sequenced.

### 4.5. Expression of NS5 of BTV, AHSV, EHDV, and KEMV in Bacteria

The confirmed pGEX-BTV1NS5, pGEX-BTV8NS5, pGEX-KEMVNS5, pGEX-EHDV7NS5, and pGEX-AHSV4NS5 plasmids, which encode GST-NS5 fusion proteins, were used to transform C41 bacteria. For each plasmid, a single colony was grown overnight at 37 °C in 2XYT medium (16 g/L tryptone, 10 g/L yeast extract, and 5 g/L NaCl) with 100 µg/mL ampicillin, then used to seed 200 mL of fresh 2XYT medium with ampicillin. The bacteria were grown until an OD_600_ of 0.5, cooled on ice for 15 min, and then 1 mM IPTG was added for induction at 28 °C for 8 h. The bacterial cells were pelleted and processed using Bugbuster^®^ protein purification (Novagen, Reno, NV, USA) as previously described [51,78]. The soluble fraction of the fusion protein was purified by glutathione affinity chromatography using glutathione sepharose, as directed by the manufacturer (GE Healthcare, Chicago, IL, USA). The purified GST fusion proteins were analysed by sodium dodecyl sulfate/polyacrylamide gel electrophoresis (SDS-PAGE) using a 10% polyacrylamide separating gel (Miniprotean III tank—Bio-Rad, Hercules, CA, USA) with a 3% stacking gel and stained with Coomassie brilliant blue, as described previously [51]. The purified proteins were also used to immunise Balb/c mice (Charles River, Wilmington, MA, USA) with an initial injection, followed by two boosts at two weeks intervals, in the presence of Montanide ISA50V2 (Seppic, Courbevoie, France) as an adjuvant. For biochemical analyses, the purified proteins were treated with 1 M NaCl to remove potential bacterial nucleic acids bound to them.

### 4.6. Western Blot Analysis of Infected Cell Cultures

Pellets of BTV-infected BSR cells (5 × 10^6^ cells) or KC cells (5 × 10^6^ cells) were dissolved by boiling for 10 min in 1 mL of sample denaturation buffer (160 mM Tris-HCl, 4 mM EDTA, 3.6% SDS, 60 mM DTT, 0.2% β-mercaptoethanol, 0.8% methionine, 800 mM sucrose). A volume of 20 µL was analysed per well by SDS-PAGE using a 10% polyacrylamide minigel (Miniprotean III tank—Bio-Rad). The resolved proteins were electro-blotted onto a 0.2 µm nitrocellulose membrane (Bio-Rad) using 20 mM Tris, 0.05% SDS, 150 mM glycine, and a 20% vol/vol isopropanol transfer buffer. Membranes were blocked with 5% skimmed milk in Tris-buffered saline (TBS: 25 mM Tris/HCl, 150 mM NaCl, 2 mM KCl, pH 7.4).

Mouse anti-sera to the different GST-fused NS5 proteins were pre-adsorbed onto non-infected BSR cells to remove cell-specific antibodies before being used in Western blot or confocal microscopy. Briefly, the sera were diluted with an equal volume of PBS and adsorbed by 3 successive incubations (each lasting 30 min) onto monolayers of non-infected BSR cells in 12-well plates. Blocked membranes were incubated overnight with a dilution of 1/300 of the mouse antiserum. Membranes were washed three times with TBS-Tween-20 (TBS containing 0.05% Tween-20) and further incubated with monoclonal anti-mouse peroxidase conjugate (Sigma), diluted at 1/750 in 5% skimmed milk. After 2 h, the membrane was washed three times with TBS-Tween-20 and developed using ECL (Biorad) in the presence of hydrogen peroxide.

### 4.7. Localisation of NS5 in Orbivirus Infected Cells by Confocal Fluorescence Microscopy

BSR cells were grown on coverslips placed within the wells of the cell culture plate. When cells reached 50% confluence, they were infected with 0.1 pfu/cell of BTV-1RSA, BTV-8, EHDV-7, or ASHV-4 and incubated at 37 °C for 24 h post-infection. Cells were then fixed in 4% paraformaldehyde and processed for immunofluorescence by permeabilizing using Triton-X100. Pre-adsorbed mouse anti-sera raised against GST-fused NS5 of BTV, AHSV, EHDV, or KEMV and rabbit anti-fibrillarin antibodies (Sigma), were diluted 1/500 in PBS containing 0.5% bovine serum albumin (PBS-A) and applied to the infected and fixed cells. After an incubation for 1 h at room temperature (RT), slides were washed in PBS and then incubated with Alexa Fluor 488 conjugated anti-mouse IgG and/or Alexa Fluor 568 conjugated anti-rabbit (Invitrogen, Waltham, MA, USA), both diluted to 1/250 in PBS. After labelling with primary and secondary antibodies, the cells were stained with DAPI (1/10,000) for 15 min for nuclear staining and mounted with Vectashield (Vector Laboratories, Newark, CA, USA) for confocal microscopy.

### 4.8. Localisation of Recombinant Expressed BTV-NS5 by Fluorescence Microscopy in Cells Transfected with pCI-BTV1NS5-GFP, pCI-BTVNS4/pCI-BTV1NS5 or pCI-BTV1NS5-GFP/VP5

BSR cells grown in 24 well plates (75% confluence) were transfected in triplicate with pCI-BTV1NS5-GFP, pCI-BTVNS4/pCI-BTV1NS5, or pCI-BTV1NS5-GFP/VP5 (4 µg/well) using Lipofectamine 3000 as described by the manufacturer (Invitrogen). Cells were fixed at room temperature for 1 h in 4% paraformaldehyde and processed for immunofluorescence, using anti-NS5, anti-NS4, or anti-VP5 antibodies, as described above.

### 4.9. Localisation of NS5 to Mitochondria

Cells infected with BTV-1 or AHSV-4 for 24 h were stained with MitoTracker^®^ Red CMXRos (Invitrogen, prepared according to the manufacturer’s recommendations) diluted in the culture medium to a final concentration of 100 nM. The cells were incubated in presence of a Red CMXRos probe for 30 min. Cells were washed twice with PBS, fixed with 4% paraformaldehyde, and permeabilized with 0.1% Triton-X100. Mouse anti-NS5 antibodies were used to identify NS5 expression and distribution in infected cells together with Alexa Fluor 488-conjugated anti-mouse IgG (Invitrogen).

### 4.10. Pulldown of Protein–Protein Complexes Formed in BSR Cells Expressing 6xHis-Tagged NS5

BSR cells were grown in 12-well plates (10^5^ cells/well). Twenty-four hours later, cells were transfected with plasmids pCI-BTV1NS5-6xHis or pCI-AHSVNS5x6His (1 µg each) using Lipofectamine 3000. At 48 h post-transfection, cells were harvested, pelleted at 500× *g*, and washed twice with ice-cold PBS at 4 °C. Cell pellets were treated with RIPA buffer (50 mM Tris-HCl pH 8.0, 150 mM NaCl, 10% NP-40, 1% sodium deoxycholate, and 0.1% SDS) containing an EDTA-free antiprotease cocktail for 30 min with continuous rotation. The lysates were centrifuged at 13,000× *g* for 10 min at 4 °C. Clarified lysates were stored on ice.

For pulldown, the Dynabeads^®^ His-Tag Isolation and Pulldown kit were used as described by the manufacturer. Briefly, 50 µL of magnetic bead suspension was transferred into a 1.5 mL microcentrifuge tube. The ethanol bead storage solution was removed after incubating the tube on the magnet for 2 min. The beads were washed four times with 300 μL 1× binding/wash buffer. Clarified transfected-cell lysates were diluted (vol/vol) in 2× pulldown buffer (6.5 mM sodium phosphate, pH 7.4, 140 mM NaCl, 0.02% Tween-20), then mixed with the beads and incubated with rotation at 4 °C for 10 min. The magnetic beads were then pulled down magnetically, and the supernatant was discarded. The beads were then washed four times with 300 μL 1× binding/wash buffer. NS5/host-cell protein–protein interactions were assessed by the addition of 3 × 100 µL elution buffer (300 mM imidazole, 50 mM sodium phosphate pH 8.0, 300 mM NaCl, 0.01% Tween-20) and analysis of the eluted protein complexes by SDS-PAGE and Western blotting.

### 4.11. Generation of BTV-1 Deletion Mutants (ΔNS4, ΔNS5 and ΔNS4/ΔNS5) by Reverse Genetics Using the BTV-1RG_C7_ Backbone

The rescue of BTV-1 in BSR cells from synthetic ssRNAs has previously been described [67]. A BTV-1RG_C7_ genetic backbone was used to engineer viruses with deletion mutations for NS4, and NS5 and a double deletion mutant for NS4 and NS5. The plasmid used to generate BTV-1ΔNS5 (containing NS5 deletion mutations) was prepared by PCR amplification of the plasmid pGEX-4T-2 (which contains a full-length cDNA copy of BTV-1 Seg-10 [67]). Primers containing deoxy-uracil nucleotides replacing the deoxy-thymidine nucleotides (Table S2) were designed to mutate positions 109 (T to C) and 136 (T to C), thus changing both in-frame ATG codons to ACG (Figure S24). These mutations had no impact on the amino acid sequence of NS3, encoded by the overlapping ORF. The resulting PCR product was treated with uracil-DNA glycosylase (which cleaves uracil moieties within the primers), thus generating cohesive ends that were ligated with the thermostable Pfu ligase (Agilent Technologies). The resulting circular product was used to transform XL1-Blue bacteria (Agilent).

To rescue the NS4 deletion mutant (BTV-1ΔNS4), a synthetic plasmid of genome Seg-9 was prepared (Life Technologies, Carlsbad, CA, USA), in which all 8 in-frame ATG codons in the NS4 ORF (positions 182-184, 242-244, 248-250, 323-325, and 338-340 of Seg-9) were mutated to ACG. None of these changes affected the amino acid sequence of VP6, encoded by the overlapping ORF.

Single (BTV-1ΔNS4 or BTV-1ΔNS5) and double (BTV-1ΔNS4/ΔNS5) deletion mutants were rescued by transfecting BSR cells with the relevant ssRNAs, as previously described [67,79].

### 4.12. Replication of BTV-1RG_C7_, BTV-1ΔNS5, BTV-1ΔNS4 or BTV-1ΔNS5/ΔNS4 in Mammalian Cells

BSR cells were used to seed 24 well plates (10^5^ cells/well) in DMEM with 1% FBS. Cells were infected with BTV-1RG_C7_, BTV-1ΔNS4, BTV-1ΔNS5, or BTV-1ΔNS4/ΔNS5 at a multiplicity of infection of 0.05 pfu/cell. At 24 h post-infection, cells were harvested and pelleted by centrifugation at 2000× *g* for 10 min at 4 °C. Cell pellets were either used for RNA extraction with TRIzol or suspended in 2 mL of 18 MΩ water and subjected to 10 strokes in a Dounce homogenizer to assist cell lysis. Lysates were transferred into a 50 mL falcon tube, with 8 mL of serum-free DMEM. A volume of 10 mL of Vertrel XF (Sigma) was added, and the mixture was vigorously shaken to dissociate virus particles from cell debris [51,80], then centrifuged at 2000× *g* for 10 min at 4 °C. The supernatant was used for virus titrations by plaque assays, as previously described [81]. The RNA extracted from pelleted cells was further purified by precipitation in 2M LiCl to remove ssRNA. The purified genomic dsRNA was quantified by real-time RT-PCR with Seg-10-derived primers (Table S2) using a probe-based assay [82,83].

### 4.13. Pulldown of NS5-RNA Complexes from BSR Cells Expressing 6xHis-Tagged NS5 of BTV or AHSV, and Infected with BTV-1RG_C7_ or BTV-1ΔNS5

BSR cells were grown in DMEM supplemented with 10% FBS in 12-well plates seeded with 10^5^ cells per well. Cells were then transfected with pCI-BTV1NS5-6xHis or pCI-AHSVNS5-6xHis. At 24 h post-transfection, cells were infected with 0.1 pfu/cell of BTV-1RG_C7_ or BTV-1ΔNS5, incubated at 37 °C for another 24 h under 5% CO_2_, then washed twice with cold PBS before being processed for pulldown as described above. The expressed 6xHis-tagged NS5 proteins were pulled down using magnetic beads, washed with 1× binding/wash buffer, and RNA was extracted using the RNeasy mini kit (Qiagen), to assess RNA-NS5 interactions. Briefly, the beads are suspended in 600 µL of RLT buffer and incubated at room temperature for 5 min. The beads were pulled down using a magnet, and the supernatant was transferred into a new microfuge tube. This supernatant was then mixed with 600 µL of ethanol, and RNA purification was performed as described by the manufacturer in the RNeasy kit protocol. The extracted RNAs were reverse transcribed using Superscript III reverse transcriptase (Thermo Fisher) in the presence of random hexaprimers (Roche), as previously described [84]. The cDNAs were then tested by real-time PCR with the QuantiTect SYBR Green PCR Kit (Qiagen) using primer pairs targeting genome segments 1, 3, and 7 (Table S2).

### 4.14. Protein Synthesis in BSR Cells Infected with BTV-1RG_C7_ or BTV-1ΔNS5

BSR cells in 24 well plates (10^5^ cells/well) were infected for 2 h with BTV-1RG_C7_, BTV-1ΔNS5, or BTV-1ΔNS4/ΔNS5 at a multiplicity of 0.5 pfu/cell. The cells were subjected to puromycin labelling, as previously described [37,38]. Briefly, at 9 or 24 h post-infection, cells were pulse-labelled with puromycin (10 mg/mL final concentration) for 15 min and harvested 3 h later. The cell monolayers were washed three times with cold PBS and then dissociated by trypsin/EDTA treatment. RNA was extracted from a fraction of the cells (4 × 10^4^ cells), treated with 10 units of RNase-free DNase I, and then subjected to purification using RNeasy columns. Another fraction of the cells (4 × 10^4^ cells) was washed with cold PBS and immediately dissolved by boiling in 100 µL of protein-sample denaturation buffer.

PCR primers were designed from the sequence of the cytochrome oxidase I gene (Table S2) of the golden hamster (accession number: NC_013276, PCR amplicon size = 156 bp).

RNA extracts from the puromycin-labelled cells were subjected to reverse transcription using superscript III reverse transcriptase and random hexaprimers. The cDNA generated was tested by real-time PCR using the QuantiTect SYBR Green PCR Kit (Qiagen), and this test was used to normalise loading volumes of the protein cell lysates. Aliquots of 15–18 µL of these samples were analysed by 10% SDS-PAGE and then transferred onto nitrocellulose membranes, which were blocked with 5% (*w*/*v*) skimmed milk (in TBS, containing 0.1% Tween-20: TBST). Membranes were incubated overnight at 4 °C with a mouse anti-puromycin monoclonal antibody (clone 12D10, Sigma), diluted to 1/5000 in 5% skim milk in TBST. The membranes were then washed with TBST and further incubated for 1 h at room temperature with an HRP-conjugated anti-mouse antibody (Sigma) diluted to 1/5000 in 5% skimmed milk in TBST. The puromycilated proteins were detected by chemiluminescence using ECL reagent (Bio-Rad).

### 4.15. Transfection of BSR Cells with pCI-BTV1NS5-6xHis or pCI-AHSVNS5-6xHis and Infection with BTV-1RG_C7_ or BTV-1ΔNS5

BSR cells, grown in 12 well plates seeded with 10^5^ cells per well, were transfected with pCI-BTV1NS5-6xHis or pCI-AHSVNS5-6xHis. At 24 h post-transfection, cells were infected with 0.1 pfu/cell of BTV-1RG_C7_ or BTV-1ΔNS5, incubated at 37 °C for 24 h under 5% CO_2_, then harvested, washed twice with ice-cold PBS, and pelleted at 500× *g* at 4 °C. The cell pellets were treated with RIPA buffer (50 mM Tris-HCl pH 8.0, 150 mM NaCl, 10% NP-40, 1% sodium deoxycholate, and 0.1% SDS) containing an EDTA-free antiprotease cocktail, for 30 min with continuous rotation. Cell lysates were subsequently centrifuged at 13,000× *g* for 10 min at 4 °C, and the clarified lysates were stored on ice. A Dynabeads^®^ His-Tag isolation and pulldown kit was used as described by the manufacturer. Briefly, 50 µL of magnetic bead suspension was transferred into a 1.5 mL microcentrifuge tube. The magnetic beads were pulled down by incubating on the dynamagnet for 2 min and the ethanol storage solution was removed. The beads were washed four times with 300 μL 1× binding/wash buffer. Clarified lysates were diluted (*v*/*v*) in the 2× pulldown buffer (6.5 mM sodium phosphate, pH 7.4, 140 mM NaCl, 0.02% Tween-20), mixed well with the beads, and incubated with rotation at 4 °C for 10 min. The magnetic beads were pulled to the wall of the tube using a magnet, the supernatant was discarded, and the beads were washed four times with 300 μL 1× binding/wash buffer. Protein complexes were eluted from the beads by the addition of 3 × 100 µL of elution buffer (300 mM imidazole, 50 mM sodium phosphate pH 8.0, 300 mM NaCl, 0.01% Tween-20) and analysed by electrophoresis and Western blotting. Alternatively, the beads were washed with 1× binding/wash buffer and an RNeasy mini kit (Qiagen) was used to extract RNA as already described.

### 4.16. Electromobility Shift Assays (EMSA)

The soluble fractions of GST-fused NS5 of BTV-1, AHSV-4, or KEMV expressed in bacteria were used in electromobility gel shift assays (EMSA) to assess their nucleic acid binding potential. Binding reactions were performed by incubating 100 ng of nucleic acids (DNA or dsRNA) for 30 min with 100 ng of purified NS5 in 0.5× Tris-borate buffer (0.5× TBE: 45 mM Tris-borate, 1 mM EDTA) containing 100 mM NaCl at room temperature. The nucleic acids used in these assays included the pCIneo plasmid and pCIneo containing a synthetic insert of 24 nucleotides consisting of twelve repetitions of the CG dinucleotide. The same plasmids were also linearized by digestion with HindIII and used in EMSA. The effect of divalent cations on binding was assessed by adding 5 mM MgCl_2_, CaCl_2_, or ZnCl_2_ into the binding reaction. ATP (150 µM) and/or DTT (1 mM) were also included in several assays to assess their effect on the binding of NS5 to nucleic acids. After incubation, binding reactions were mixed with a loading buffer (containing 2.5% Ficoll-400, 10 mM EDTA, 0.08% SDS, and 3.3 mM Tris-HCl pH 8.0), and 0.1 µg of ethidium bromide was added. Nucleic acid-protein complexes were resolved on a non-denaturing 1.5% agarose in ice-cold TBE buffer containing 100 mM NaCl. Electrophoresis was performed at 50 V for 90 min and nucleic acids were visualised by UV transillumination.

### 4.17. Cloning of NS5 ORF in a Vaccinia Shuttle Vector, Generation of Vaccinia-ΔE3L/+NS5 Recombinant Viruses and Replication in Interferon Treated Cells

The NS5 ORF of BTV-1 and the ORF of E3L were inserted into synthetic shuttle plasmids (ShuttleVacc) by restriction cloning using the enzyme BsaI. The shuttle allows the insertion of transgenes at the E3L locus of the vaccinia virus Copenhagen (VVC) by homologous recombination, thereby substituting E3L with the transgene of interest. The ShuttleVacc plasmid, prepared in the pMA vector (GeneArt, Regensburg, Germany), was designed using the nucleotide sequence of the vaccinia virus Copenhagen (accession number M35027) to contain a “right arm” of recombination spanning nucleotides 51,483 to 51,982, followed by the p11 vaccinia promoter, which drives expression of a given heterologous gene. ShuttleVacc also contains a “left arm,” containing recombination spanning nucleotides 50,438–50,914 of the vaccinia virus. A BsaI restriction site allows directional cloning of genes of interest using the Golden Gate cloning method. The ORFs encoding NS5 of BTV-1 and E3L of VV were PCR amplified with primers containing the BsaI site (Table S3) to facilitate cloning into the ShuttleVacc.

After homologous recombination, a cassette containing the transgene, driven by promoter p11 and a yellow fluorescent protein (YFP), under the control of the encephalomyocraditis virus (EMCV) IRES, was transferred into VVC-ΔE3L (strain VP1080). YFP expression facilitated the identification of the recombinant vaccinia virus clones. Homologous recombinations between VP1080 [59,60] and ShuttleVacc-NS5BTV, or ShuttleVacc-E3L, were performed in chicken embryo fibroblasts, as previously described [59]. The insertion of the NS5 or E3L ORF was confirmed by PCR (using cloning primers shown in Table S3) performed on DNA extracted from the purified recombinant plaques, expanded in DF1 cells. Recombinant vaccinia viruses (VV-VP1080-BTV1NS5 and VV-VP1080-E3L) were subjected to three rounds of plaque purification. Fluorescent plaques were recovered and further propagated in chicken embryo fibroblasts (Figure S25).

Monolayers of A549 (human lung carcinoma cells) were grown in 25 cm^2^ culture flasks. Cells were infected with an MOI of 10 pfu of wtVV Copenhagen, VV-VP1080-BTV1NS5, or VV-VP1080-E3L per cell. At 24 h post-infection, cells were harvested, and RNA was extracted from cell pellets using TRIzol. The integrity of 28S and 18S ribosomal RNA was assessed by agarose gel electrophoresis using a 1% agarose gel in 1X TAE buffer (40 mM Tris, 20 mM acetic acid, 1 mM EDTA, pH 8.0).

## Figures and Tables

**Table 2:** Comparisons of real-time PCR Ct values obtained for RNA extracts of pulldown complexes from non-transfected cells and those ectopically expressing NS5-6xHis.

(a)							
Infection with BTV-1ΔNS5		Normalized Mean Ct Values for Non-Transfected (Ctrl) and Transfected Cells			Standard Deviation		
Experiment	Viral RNA Detected	Ctrl	NS5 (AHSV)	NS5 (BTV)	Ctrl	NS5 (AHSV)	NS5 (BTV)
I	BTV-1 Seg-1	18.09	11.61	10.94	0.16	0.06	0.03
	BTV-1 Seg-3	19.87	11.79	11.46	0.28	0.03	0.15
	BTV-1 Seg-7	16.39	10.94	10.25	0.16	0.08	0.08
II	BTV-1 Seg-1	14.81	11.28	10.80	0.14	0.07	0.19
	BTV-1 Seg-3	19.32	11.87	11.16	0.41	0.12	0.09
	BTV-1 Seg-7	16.17	11.76	11.05	0.18	0.06	0.04
**(b)**							
**Infection with BTV-1RG_C7_**		**Normalized Mean Ct Values for Non-Transfected (Ctrl) and Transfected Cells**			**Standard Deviation**		
**Viral RNA Detected**		**Ctrl**	**NS5 (AHSV)**	**NS5 (BTV)**	**NS5 (AHSV)**	**NS5 (BTV)**	
BTV-1 Seg-1		17.80	17.60	18.01	0.82	0.08	
BTV-1 Seg-3		17.02	16.17	15.86	0.36	0.13	
BTV-1 Seg-7		18.63	17.24	17.02	0.16	0.18	

(a): All of the cells had been infected with BTV-1ΔNS5. The values shown are the means of triplicates. The experiment was repeated on two separate occasions. Differences of 3.5 to 8.4 Cts were detected between the transfected and non-transfected cells. (b): All of the cells had been infected with BTV-1RG_C7_. The values shown are the means of triplicates. There are no significant differences between control (Ctrl) cells and those expressing NS5-6xHis.

## Data Availability

Data are presented in the manuscript and associated Supplementary Materials.

## Supplementary Materials

The following supporting information can be downloaded at: https://www.mdpi.com/article/10.3390/ijms24076845/s1.
