# Supplementary material for: Identification of Orbivirus Non-Structural Protein 5 (NS5), Its Role and Interaction with RNA/DNA in Infected Cells
Source: Int J Mol Sci. 2023 Apr 6;24(7):6845. doi: 10.3390/ijms24076845 (PMC10095184; doi:10.3390/ijms24076845)
Supplement: Supplementary file 1 [file ijms-24-06845-s001.zip › NS5 paper supplementary data after conversion.pdf]

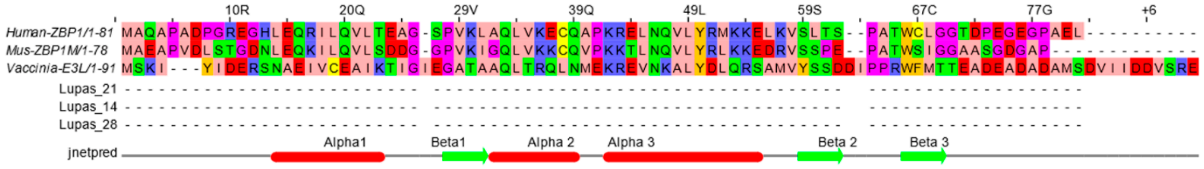

Figure S1: alignment and secondary structure prediction of human or mouse ZBP1 and VV E3L showing the previously described alpha1-beta1-alpha2-alpha3-beta2-beta3 organisation.

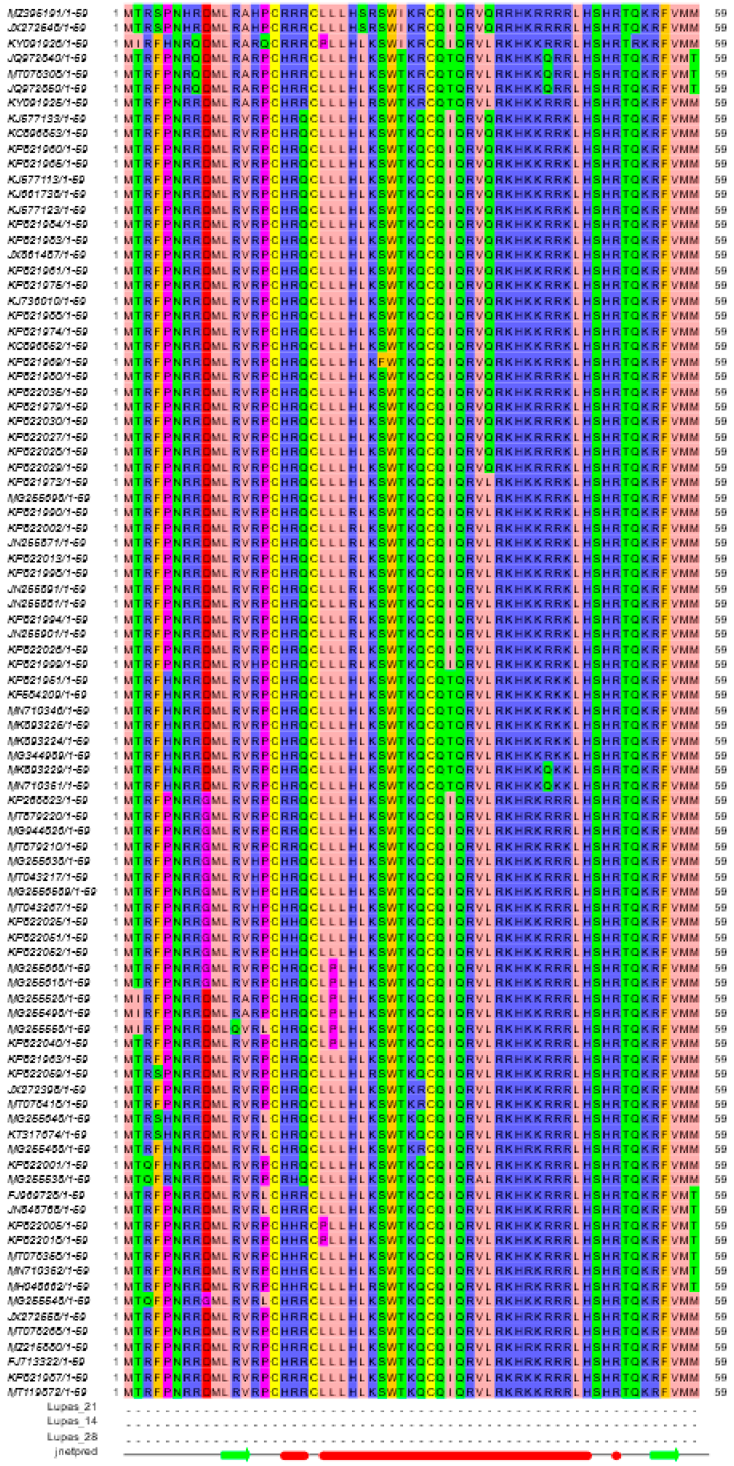

Figure S2: Prediction of BTV NS5 secondary structure using JPred.

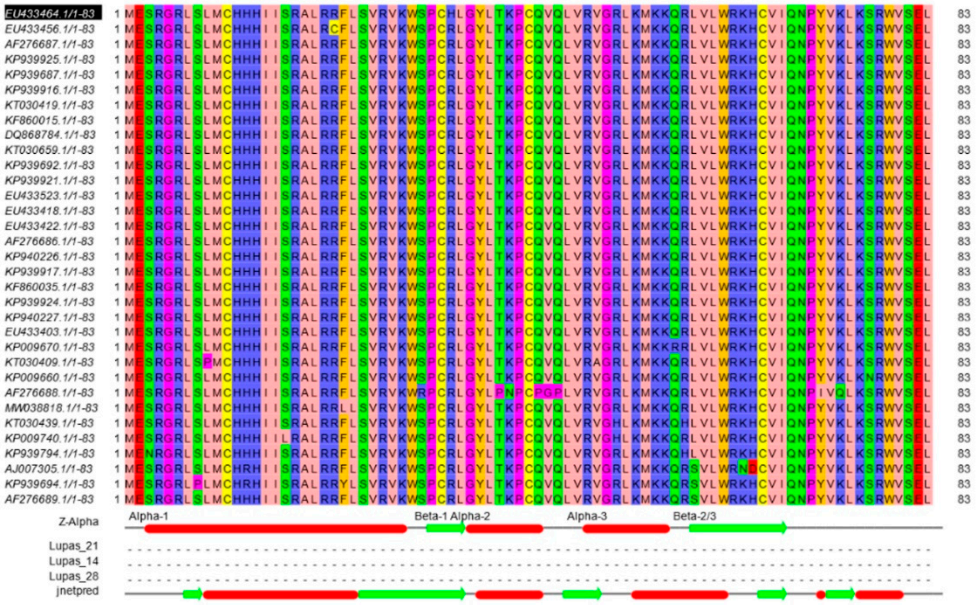

Figure S3: alignment of AHSV NS5 sequences and prediction of secondary structure by JPred. The predicted structure is globally similar to the Z-alpha domain of ZBP1 and vaccinia virus E3L.

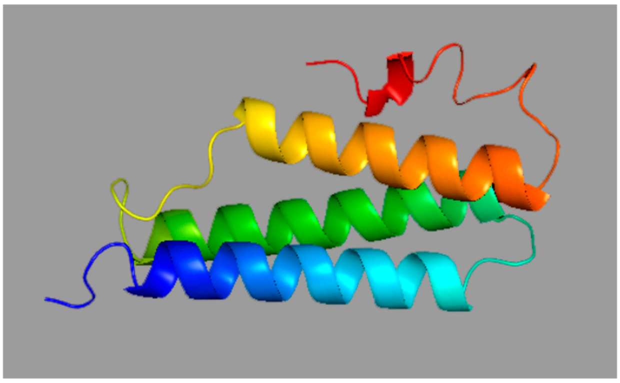

Figure S4: Predicted structure of NSP6 using Raptor. The predicted structure is mainly alpha helical.

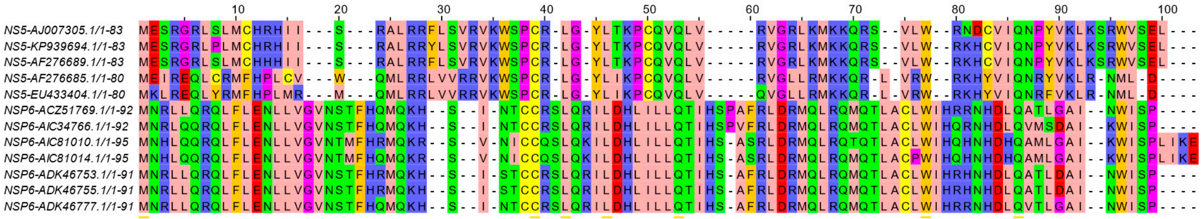

Figure S5: Alignment of representative AHSV NS5 sequences with rotavirus A NSP6. The two proteins have similar sizes. The two proteins show conserved residues/motifs, suggesting that they may represent orthologues.

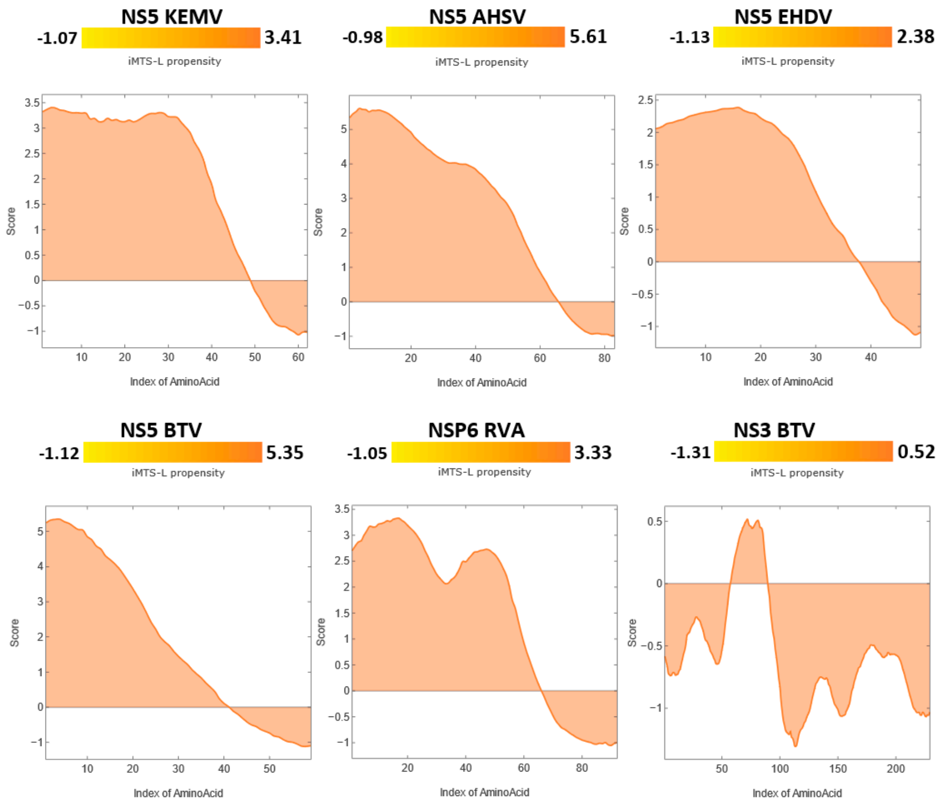

Figure S6: iMTS-L scores and propensity heatmap showing a high potential for orbivirus NS5 sequences to localise into the mitochondrial matrix. Similar scores are identified for rotavirus A NSP6. Previous studies (immunofluorescence) concluded that NSP6 localises to the mitochondria, which is also predicted in the current study by iMLP. The potential localisation of orbivirus NS5 to mitochondria is assessed in the current study by immunofluorescence analyses.

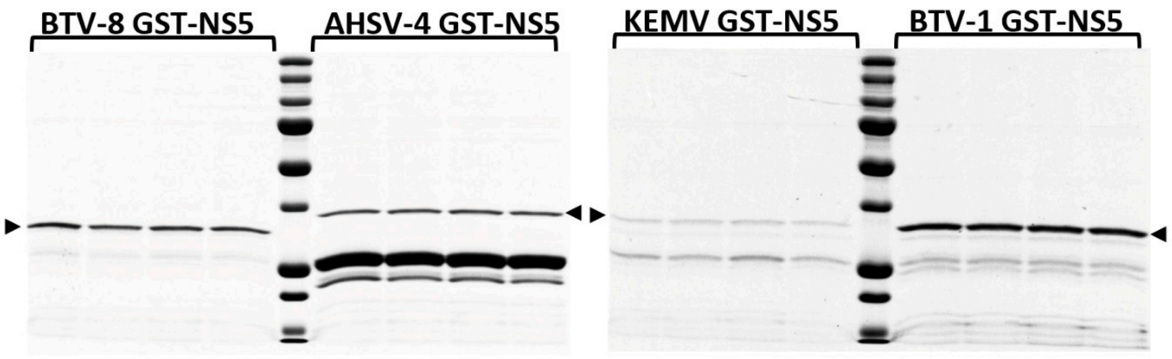

Figure S7: Glutathione affinity purified GST-fused NS5 of BTV-1, BTV-8, AHSV-4 and KEMV. The proteins were analysed by PAGE on a 10% polyacrylamide gel and stained with Coomassie blue. The full-length fusion protein is indicated by an arrow Some degradation is visible below the full-length protein.

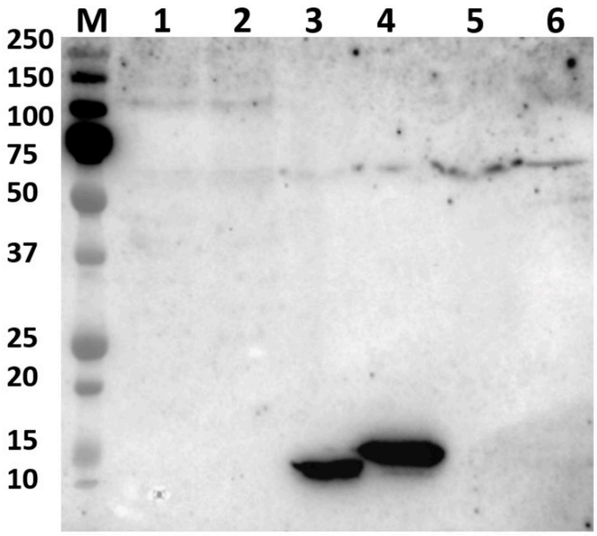

Figure S8: Western blot analysis of pulldown products from BSR cells expressing his-tagged NS5 of BTV-1 or AHSV-4.

Anti-histag antibodies were used detecting his-NS5 of BTV-1 or AHSV-4 with high sensitivity. Non-specific protein bands were also detected in both transfected and non-transfected cells but with significantly lower sensitivity. Lane M: size marker labelled in kDa, lane 1 and 2: total BSR cell lysate, lane 3: pulled BTV-1 NS5, lane 4: pulled AHSV-4 NS5, lanes 5 and 6: pulled non-transfected BSR lysates.

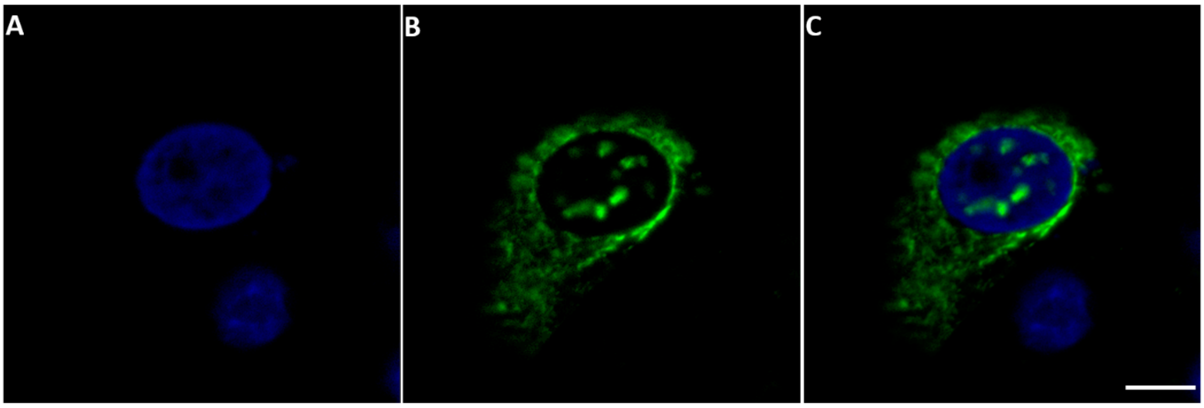

Figure S9: Confocal immunofluorescence microscopy analysis of NS5 expression in BTV-1 infected BSR cells.
Anti-NS5 antibodies identify NS5 both in the cytoplasm and to a lesser extent in the nucleus. Mouse serum was adsorbed onto non-infected BSR cells to remove any potential non-specific antibodies. **A:** nuclei coloured by DAPI, **B:** NS5 signal identified by mouse anti-NS5 of BTV-1 and revealed by Alexa Fluor 488 (green fluorescence) conjugated anti-mouse IgG, **C:** a merge of A and B panels. Scale bar represents 5µm.

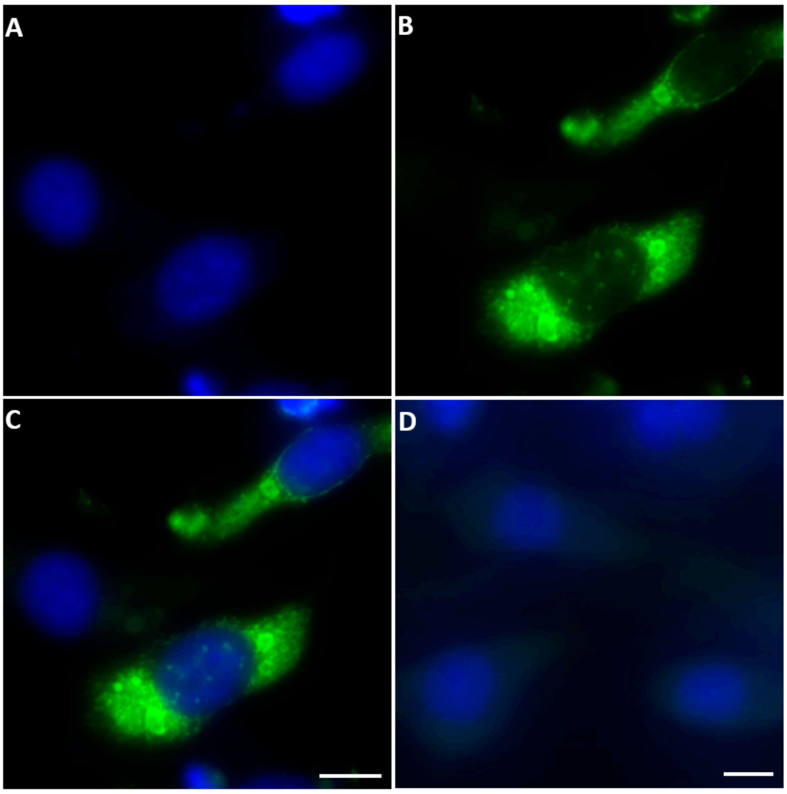

Figure S10: Immunofluorescence microscopy analysis of NS5 expression in BTV-8 RSArrrr/08 infected BSR cells.
Anti-NS5 antibodies identified NS5 both in the cytoplasm and at a lower level in the nucleus of BTV-8 infected BSR cells. **A:** nuclei coloured by DAPI, **B:** NS5 signal identified by anti-BTV-8 NS5 antibodies and revealed by Alexa Fluor 488 (green fluorescence) conjugated anti-mouse IgG, **C:** a merge of A and B panels and **D:** non-infected BSR cells tested with anti-BTV-8 NS5 antibodies and Alexa Fluor 488 (green fluorescence) conjugated anti-mouse IgG (negative for NS5 detection). Scale bar represents 5µm.

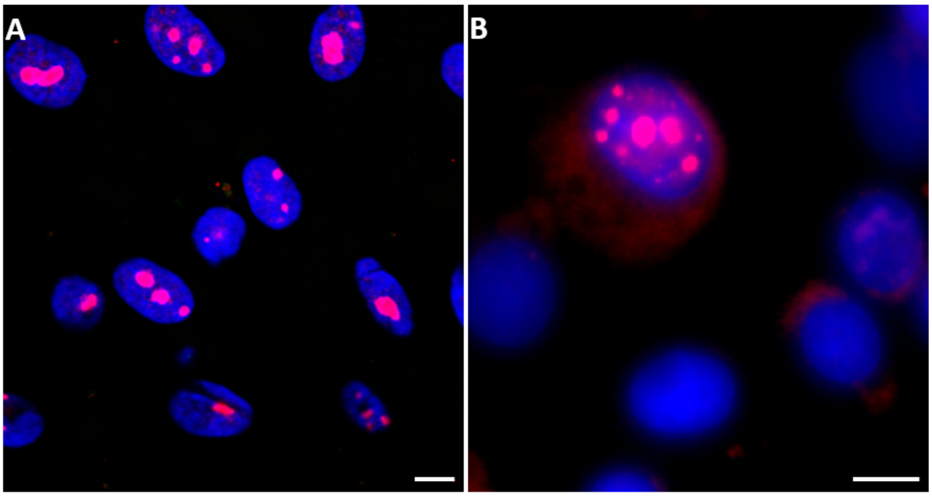

Figure S11: Immunofluorescence microscopy showing NS5 expression in BTV-1ΔNS5 infected BSR cells.
Immunofluorescence microscopy using pre-adsorbed mouse anti-NS5 serum and Alexa Fluor 488 conjugated anti-mouse IgG, did not detect NS5 in infected BSR cells (result not shown). **A:** BTV-1ΔNS5 infected cells probed with rabbit anti-fibrillarin antibodies and Alexa Fluor 568 conjugated anti-rabbit antibodies, revealed fibrillarin in the nucleoli (red fluorescence). **B:** BTV-1ΔNS5 infected BSR cells, probed with rabbit anti-NS4 antibodies and Alexa Fluor 568 conjugated anti-rabbit antibodies, also detected NS4 in the nucleoli and in the cytoplasm. Scale bar represents 5µm.

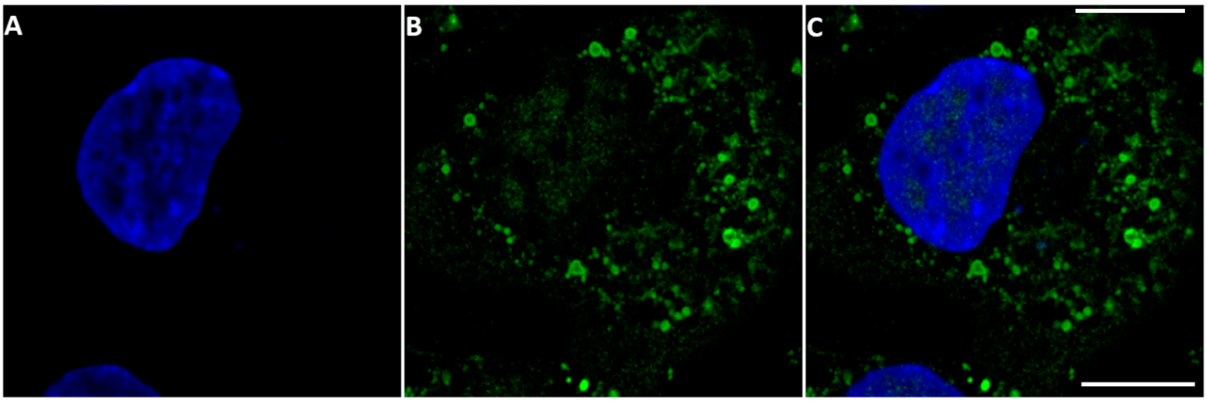

Figure S12: Confocal immunofluorescence microscopy analysis of NS5 expression in AHSV-4 morocco infected BSR cells.

Anti-NS5 of AHSV-4 antibodies identified NS5 both in the cytoplasm and in the nucleus of infected BSR cells. **A:** nuclei coloured by DAPI. **B:** NS5 signal identified by anti-NS5 of AHSV-4 antibodies and Alexa Fluor 488 (green fluorescence) conjugated anti-mouse IgG. **C:** a merge of **A** and **B** panels. Scale bar represents 5µm.

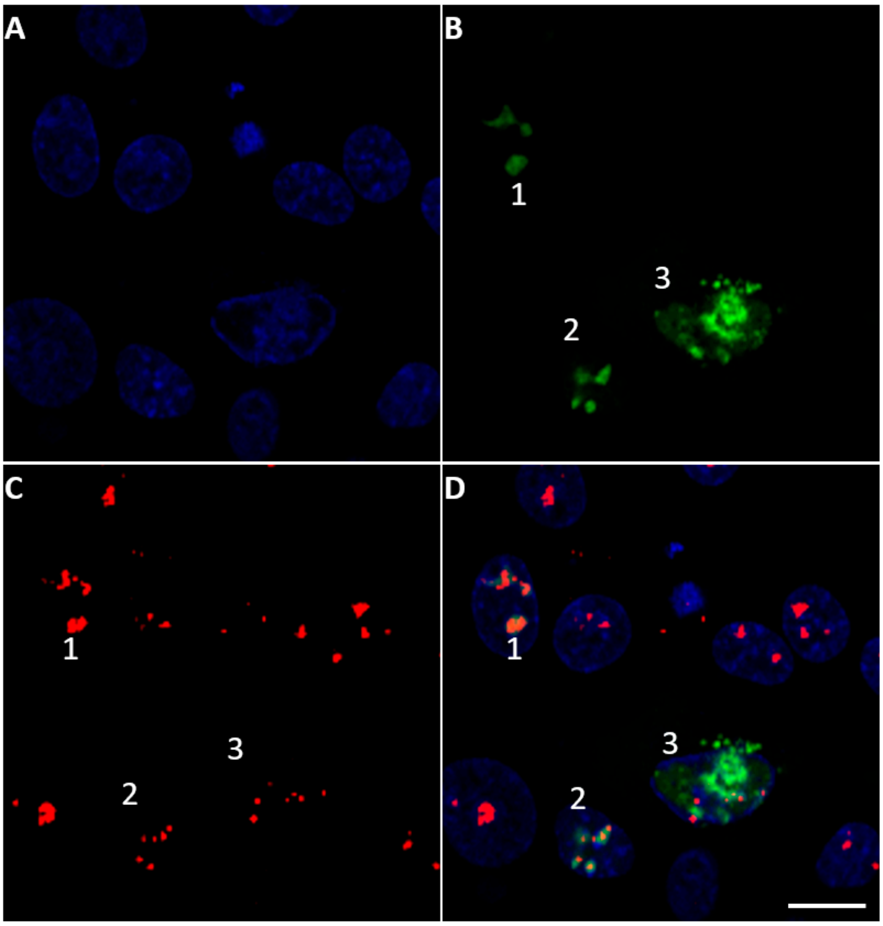

Figure S13: Detection of NS5 expression in confocal immunofluorescence microscopy of BTV-1 infected BSR cells at 16h post-infection.

Anti-BTV-1 NS5 antibodies mainly detected NS5 in the nucleus although it was also starting to accumulate in the cell-cytoplasm. **A:** cells stained with DAPI, **B:** cells probed anti-BTV-1 NS5 antibodies and Alexa Fluor 488 conjugated anti-mouse IgG (green), **C:** cells probed with anti-fibrillarin antibodies and Alexa Fluor 568 anti-rabbit IgG (red) and **D:** merge of **A**, **B** and **C.** Three infected cells (at different stages of infection) are indicated by numbers (1, 2 and 3). In cell 1, NS5 appears to localize to the nucleoli. In cell 2, smaller nucleolar structures are observed which are still associated with NS5. In cell 3, NS5 is visible across the nucleus and in the cytoplasm as well. Scale bar represents 5µm.

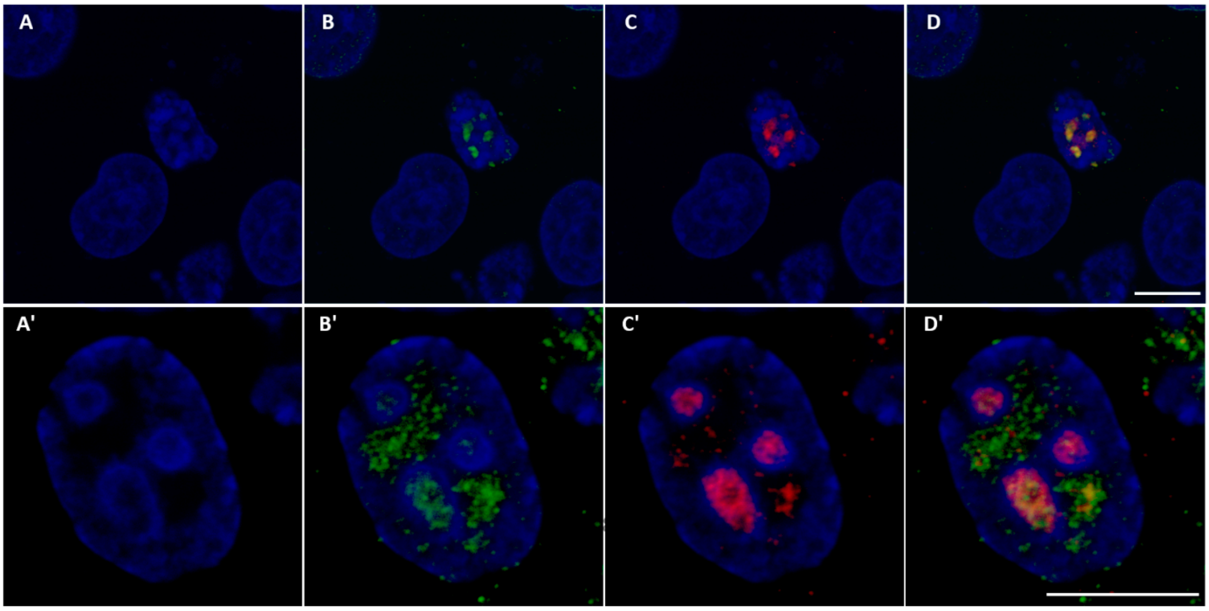

Figure S14: Confocal immunofluorescence microscopy of cells expressing NS5 and NS4 of BTV-1 at 24hrs post-transfection. **A or A':** cells were stained with DAPI, **B or B':** NS5 expression (green) visible in the nucleus as well as in the cytoplasm of certain cells and was detected using mouse anti-NS5 antisera and Alexa Fluor 488 conjugated anti-mouse IgG, **C or C':** NS4 expression (red) visible in the nucleus as well as in the cytoplasm of certain cells and was detected using rabbit anti-NS4 anti-sera and Alexa Fluor 568 conjugated anti-rabbit IgG, **D:** merge of **A , B** and **C** and **D':** merge of **A' , B**' and **C'**. Scale bar represents 5µm.

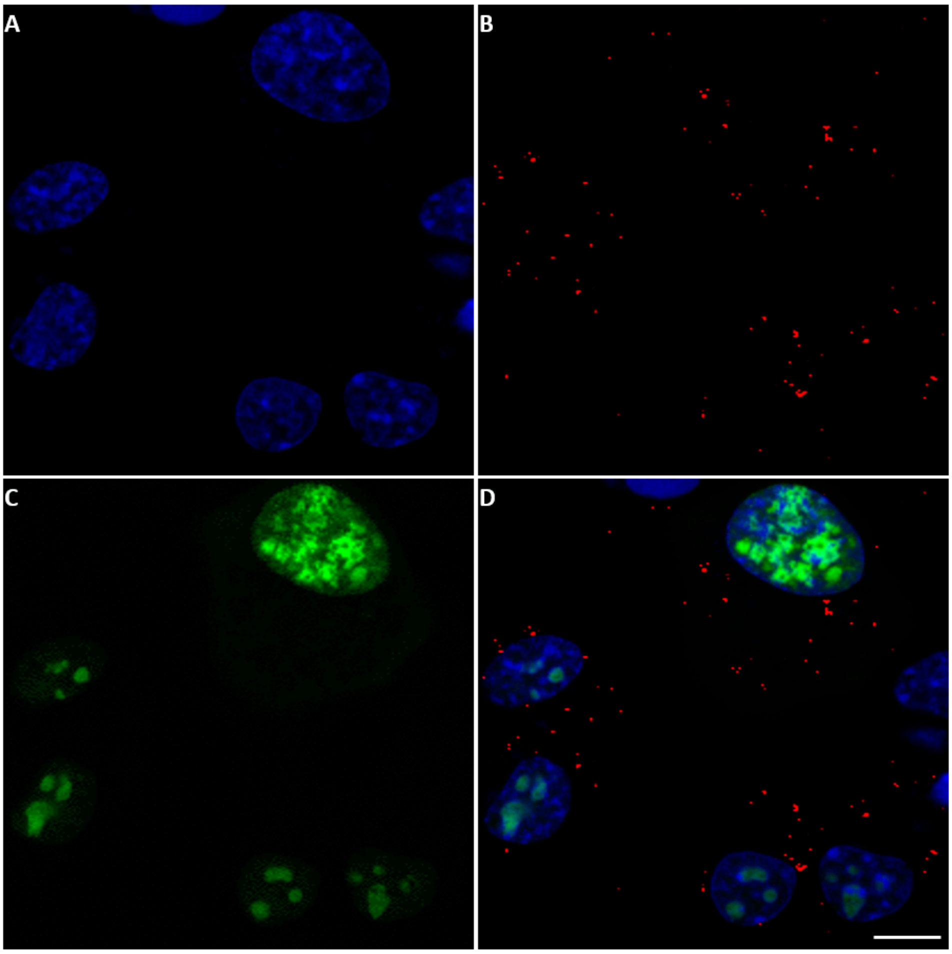

Figure S15: Confocal immunofluorescence microscopy of cells transfected with pCI-BTV1NS5/BTV4VP5, expressing NS5 and VP5 of BTV, at 24 hrs post-transfection.

A: cells were stained with DAPI, **B:** VP5 expression (red) was detected using anti-VP5 antibodies and is visible in the cytoplasm of cells, **C:** NS5-GFP (green) is mainly visible in the nucleus, and **D:** merge of **A, B** and **C**. scale bar represents 5μm.

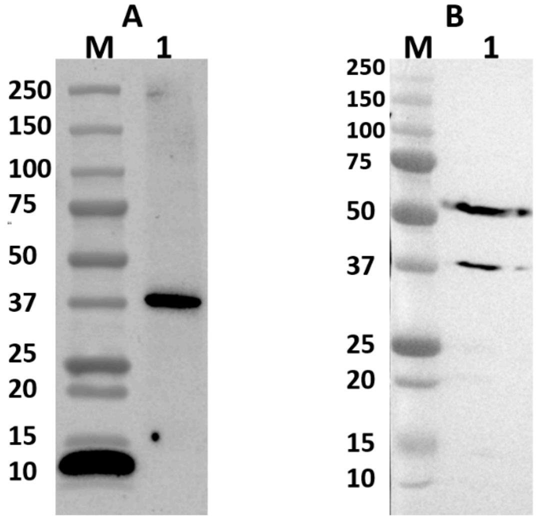

Figure S16: Western blot analysis for ZBP1 expression in Hela cells and BSR cells:

In panel A; Lane M: size marker labelled in kDa, lane 1: western blot of total HeLa cell lysate probed with antibody to human ZBP1 and showing an intense band at around 40kDa. In Panel B; Lane M: size marker labelled in kDa; lane 1: western blot of total BSR cell lysate probed with antibody to human ZBP1 showing two band at around 40kDa and 50kDa. The Genbank database contains sequences of 4 isoforms for the ZBP1 of the Syrian golden hamster (from which BSR cells are derived). These are isoform X1 (428aa, accession XP_040588843), isoform X2, (421aa, accession XP_040588844.1), isoform X3 (406aa, accession XP_005074637) and isoform X4 (402aa, accession XP_040588845). The theoretical sizes of these 4 isoforms range from 42 to 45kDa.

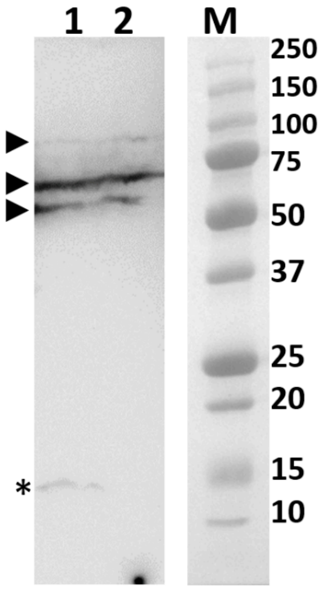

Figure S17: Western blot analysis of BSR cell lysates. BSR cells-specific bands can be observed in western blots of BSR lysates either mock transfected of transfected with pCI-BTV1NS5-6xHis. Cells were harvested at 24hrs post-transfection and washed 3 times with PBS. Cell pellets were directly dissolved in sample denaturation buffer and analysed by SDS-PAGE and western blot using anti-6xHis antibodies. Lane M: size marker labelled in kDa. Western blot analysis was performed using anti-6xHis antibodies. Cells expressing NS5 of BTV-1 (transfected with pCI-BTV1NS5-6xHis plasmid) in lane 1, show a band at ~15kDa, which is absent from non-transfected cells in lane 2. Non-specific protein bands (with sizes of ~65, 75 and 100 kDa) are clearly identified by the anti-6xHis antibodies in both lanes 1 and 2.

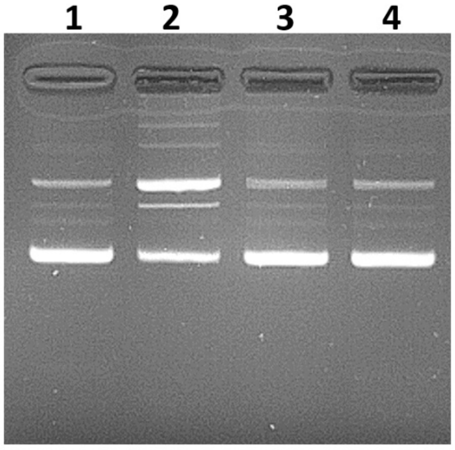

Figure S18: Electrophoretic mobility shift assays showing the effect of divalent cations on the binding of DNA to NS5 of BTV:

Protein-DNA binding reactions were performed in 0.5X TBE buffer containing 100 mM NaCl and 5 mM of $MgCl_2$, $CaCl_2$ or $ZnCl_2$, incubated for 30 minutes at RT. Products were analysed by electrophoresis on 1% agarose in 0.5X TBE containing 100 mM NaCl. Nucleic acids were visualized after electrophoresis by UV transillumination in the presence of ethidium bromide. Lane 1: pCIneo-24CG plasmid (100 ng). Lane 2: pCIneo-24CG plasmid (100 ng) plus 100 ng of BTV GST-fused NS5 in presence of $MgCl_{2.}$ Lane 3: Lane 2 pCIneo-24CG plasmid (100 ng) plus 100 ng of BTV GST-fused NS5 in presence of $CaCl_2$. Lane4: pCIneo-24CG plasmid (100 ng) plus 100 ng of BTV GST-fused NS5 in presence of $ZnCl_2$.

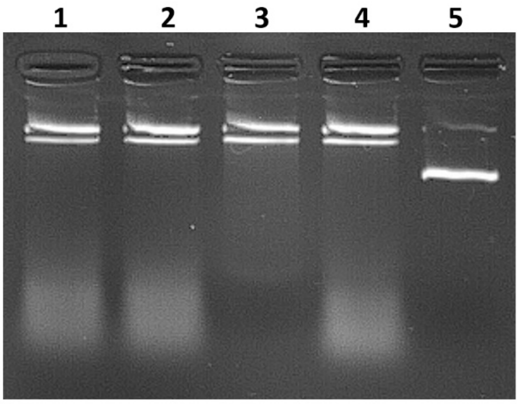

Figure S19: Electrophoretic mobility shift assays of GST-fused NS5 of AHSV
All protein-DNA binding reactions were performed in 0.5X TBE buffer containing 100 mM NaCl and 5 mM $MgCl_2$. Reactions were incubated for 30 minutes then analysed in 1% agarose gel containing TBE 0.5x and 100 mM NaCl. Nucleic acids were visualized after electrophoresis by UV transillumination in the presence of ethidium bromide. Lanes 1, 2 and 4: pCIneo-24CG plasmid (100ng) together with 100ng of AHSV GST-fused NS5, Lane 3: pCIneo-24CG plasmid (100ng) together with 5ng of AHSV GST-fused NS5 and Lane 5: pCIneo-24CG plasmid (100ng) only.

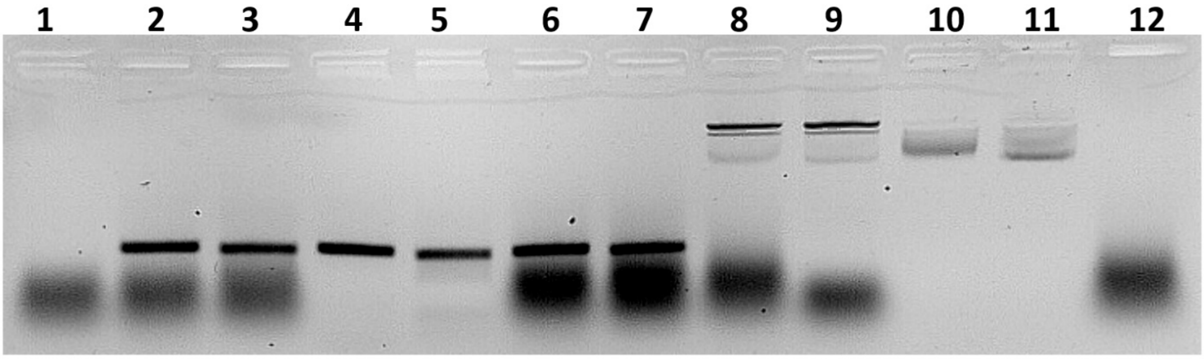

Figure S20: Electrophoretic mobility shift assays of GST-fused NS5 of BTV-8 or KEMV with linear DNA:
All protein-DNA binding reactions were performed in 0.5X TBE buffer containing 100 mM NaCl and 5 mM $MgCl_2$. Reactions were incubated for 30 minutes then analysed by electrophoresis in 1% agarose gel containing TBE 0.5x and 100 mM NaCl. Nucleic acids were visualized after electrophoresis by UV transillumination in the presence of ethidium bromide. PCR products of ~1000 bp long were used in the assays. Two distinct PCR products were used, one containing a Zeocin resistance gene and the other a ccdb. The PCR product containing Zeocin resistance gene has a mean G+C content of 52% (the zeocin sequence itself is 375 bp-long and has a G+C content of 69%). The product containing ccdb has a mean G+C content of 43% (the ccdb sequence itself is 232 bp-long and have a G+C content of 27%). Lane 1: 100ng of GST-fused KEMV NS5. Lane 2: 100ng of GST-fused KEMV NS5 in presence of 100ng of PCR product containing the zeocin sequence. Lane 3: 100ng of GST-fused KEMV NS5 in presence of 100ng of PCR product containing the ccdb sequence. Lane 4: 100ng of PCR product containing the zeocin sequence. Lane 5: 100ng of PCR product containing the ccdb sequence. Lane 6: 100ng of GST-fused BTV-8 NS5 in presence of 100ng of PCR product containing the zeocin sequence. Lane 7: 100ng of GST-fused BTV-8 NS5 in presence of 100ng of PCR product containing the ccdb sequence. Lane 8: 100ng of GST-fused BTV-8 NS5 in presence of 100ng of pCIneo plasmid (circular). Lane 9: 100ng of GST-fused BTV-8 NS5 in presence of 100ng of pCIneo-24CG plasmid (circular). Lane 10: 100ng of pCIneo plasmid (circular). Lane 11: 100ng of pCIneo-24CG plasmid (circular). Lane 12: 100ng of GST-fused BTV-8 NS5 only.

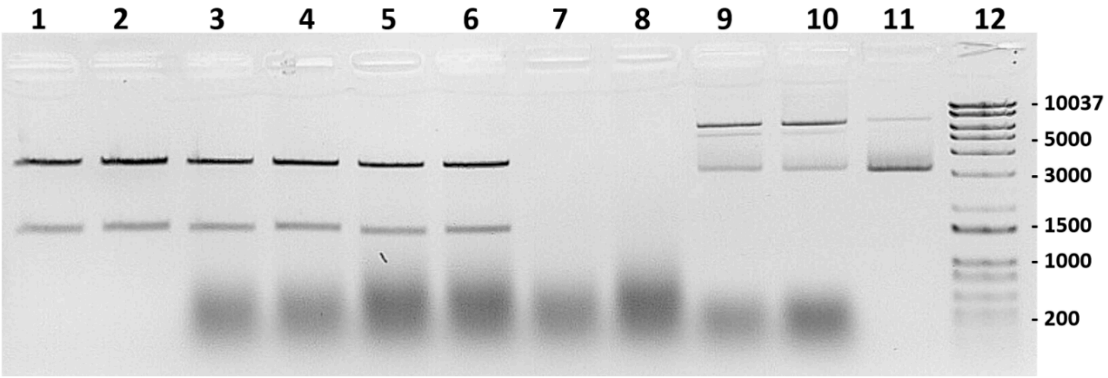

Figure S21: EMSA for GST-fused NS5 of BTV-8 or KEMV with HindIII linearised plasmids pCIneo and pCIneo-24CG.

All protein-DNA binding reactions were performed in 0.5X TBE buffer containing 100 mM NaCl and 5 mM $MgCl_2$. Reactions were incubated for 30 minutes then analysed by electrophoresis in 1% agarose gel containing TBE 0.5x and 100 mM NaCl. Nucleic acids were visualized after electrophoresis by UV transillumination in the presence of ethidium bromide. Lane 1: 100ng of cleaved pCIneo plasmid, Lane 2: 100ng of cleaved pCIneo-24CG plasmid, lane 3: 100ng of cleaved pCIneo plasmid plus 100ng of GST-fused KEMV NS5, lane 4: 100ng of cleaved pCIneo-24CG plasmid plus 100ng of GST-fused KEMV NS5, lane 5: 100ng of cleaved pCIneo plasmid plus 100ng of GST-fused BTV-8 NS5, lane 6: 100ng of cleaved pCIneo-24CG plasmid plus 100ng of GST-fused BTV-8 NS5, lane 7: 100ng of GST-fused KEMV NS5, lane8: 100ng of GST-fused BTV-8 NS5, lane 9: 100ng of circular pCIneo-24CG plasmid plus 100ng of GST-fused KEMV NS5, lane 10: 100ng of circular pCIneo-24CG plasmid plus 100ng of GST-fused BTV-8 NS5, Lane 11: 100ng of circular pCIneo-24CG plasmid only and lane 12: DNA size marker.

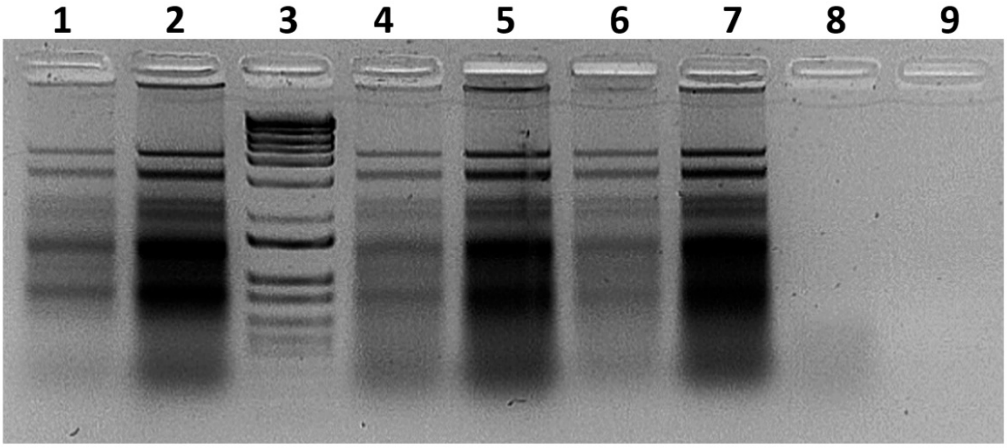

Figure S22: EMSA for GST-fused NS5 of BTV-8 with genomic dsRNA of BTV in 1% agarose gel containing TBE 0.5x and 100 mM NaCl. All protein-DNA binding reactions were performed in 0.5X TBE buffer containing 100 mM NaCl and 5 mM $MgCl_2$. Reactions were incubated for 30 minutes at RT. Lanes 1 and 2: 20 and 200ng of dsRNA, lane 3: DNA size marker, lane 4: 20ng of dsRNA plus 100ng of GST-fused BTV-8 NS5, lane 5 : 200ng of dsRNA plus 100ng of GST-fused BTV-8 NS5, lane 6: 20ng of dsRNA plus 5ng of GST-fused BTV-8 NS5, lane 7: 200ng of dsRNA plus 5ng of GST-fused BTV-8 NS5, 100ng of pCIneo-24CG plasmid only, lane 8: 100 ng of GST-fused BTV-8 NS5, lane 9: 5ng of GST-fused BTV-8 NS5.

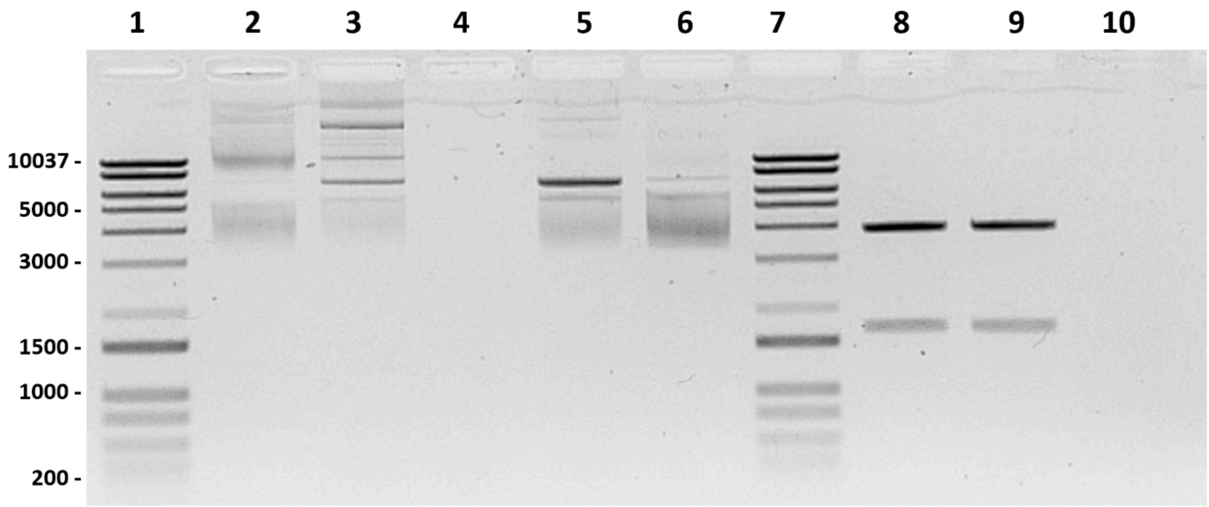

Figure S23: EMSA for GST-fused NS5 of BTV-8 with plasmids pCIneo or pCIneo-24CG
All protein-DNA binding reactions were performed in 0.5X TBE buffer containing 100 mM NaCl and 5 mM $MgCl_2$. Reactions were incubated for 30 minutes then analysed by electrophoresis in 1% agarose gel containing TBE 0.5x and 100 mM NaCl. Nucleic acids were visualized after electrophoresis by UV transillumination in the presence of ethidium bromide. Lanes 1 and 7: DNA size marker, lane 2: 100ng of pCIneo plasmid, lane 3: 100ng of pCIneo plasmid plus 5ng of GST-fused BTV-8 NS5, lane 4: 5 ng of GST-fused BTV-8 NS5, lane 5: 100ng of pCIneo-24CG plasmid plus 5ng of GST-fused BTV-8 NS5, lane 6: 100ng of pCIneo-24CG plasmid only, lane 8: HindIII cleaved pCIneo24CG (100ng), lane 9: HindIII cleaved pCIneo24CG (100ng) plus 5ng of GST-fused BTV-8 NS5, lane 10: 5ng of GST-fused BTV-8 NS5.

Wild-type Seg-10

```
                      101                 121                 141                 161
                       |                   |                   |                   |
          GAGTCTGGTCCGTGTGGATGACACGATTTCCCAACCGCCGAGATATGCTCCGAGTGCGCCTATGCCATCGTCGATGCCTACTGTTGCACT
NS3 Frame   S  L  V  R  V  D  D  T  I  S  Q  P  P  R  Y  A  P  S  A  P  M  P  S  S  M  P  T  V  A  L
NS5 Frame - V  W  S  V  W  M  T  R  F  P  N  R  R  D  M  L  R  V  R  L  C  H  R  R  C  L  L  L  H
```

Mutated Seg-10

```
                      101                 121                 141                 161
                       |                   |                   |                   |
          GAGTCTGGTCCGTGTGGACGACACGATTTCCCAACCGCCGAGATACGCTCCGAGTGCGCCTATGCCATCGTCGATGCCTACTGTTGCACT
NS3 Frame   S  L  V  R  V  D  D  T  I  S  Q  P  P  R  Y  A  P  S  A  P  M  P  S  S  M  P  T  V  A  L
NS5 Frame - V  W  S  V  W  T  T  R  F  P  N  R  R  D  T  L  R  V  R  L  C  H  R  R  C  L  L  L  H
```

Figure S24: mutation of the two in-frame ATG codons of the NS5 ORF. The nucleotide T109C and T136C mutations do not modify the amino acid sequence of NS3.

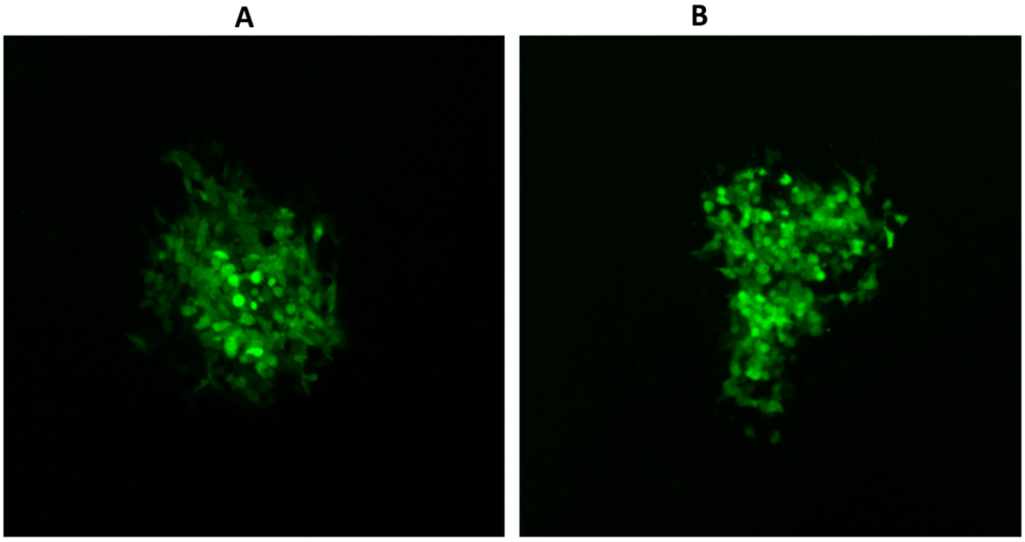

Figure S25: Fluorescent-plaques of recombinant Vaccinia virus. **A:** a fluorescent plaque of VV-VP1080-E3L. **B:** a fluorescent plaque of VV-VP1080-NS5.

Primer	Sequence (5'→3')	Restriction enzyme	Target
NS5BTV-8RSAPGEXFORECO	ctcaac**GAATTC**ATGACACGATCTCCCAACCACCG	EcoRI	NS5 BTV-8
NS5BTV-8RSAPGEXRevNot	gatgagat**GCGGCCGC**GCATCATCACGAAACGCTTCTGC	NotI	NS5 BTV-8
NS5BTV-1RSAPGEXFORECO	ctcaac**GAATTC**ATGACACGATTTCCCAACCGCCG	EcoRI	NS5 BTV-1
NS5BTV-1RSAPGEXRevNot	gatgagat**GCGGCCGC**GCGTCATCACGAAACGCTTCTG	NotI	NS5 BTV-1
NS5AHSV-4PGEXFORECO	ctcaac**GAATTC**ATGGAGAGTCGGGGGCGATTG	EcoRI	NS5 AHSV-4
NS5AHSV-4PGEXRevNot	gatgagat**GCGGCCGC**GAAGTTCTGATACCCACCTGCTTT	NotI	NS5 AHSV-4
NS5AHSV-7PGEXFORECO	ctcaac**GAATTC**ATGGAAATCAGAGAGCAATTGTGC	EcoRI	NS5 AHSV-7
NS5AHSV-7PGEXRevNot	gatgagat**GCGGCCGC**GATCCAACATGTTTCTTAATTTGACGTATCGG	NotI	NS5 AHSV-7
NS5KEMVPGEXFORECO	ctcaac**GAATTC**ATGTCGCTCTTGGCGTTCTAC	EcoRI	NS5 KEMV
NS5KEMVPGEXRe5Not	gatgagat**GCGGCCGC**GGTTTCGCCAGGGTGCGCTCCG	NotI	NS5 KEMV
NS5EHDV-7ISRPGEXFORECO	ctcaac**GAATTC**ATGTACCGTCAGCTCCAATACCCAC	EcoRI	NS5 EHDV-7
NS5EHDV-7ISRPGEXRevNoT	gatgagat**GCGGCCGC**GAATCATCTCTAAATGCCTCCGCGTACG	NotI	NS5 EHDV-7
NS5BTV8EcoFor	gattct**GAATTC**ACGATGACACGATCTCCCAACC	EcoRI	NS5 BTV-8
NS5BTV8NotREV	acatgt**GCGGCCGC***TCA*CATCATCACGAAACGCTTC	NotI	NS5 BTV-8
NS5BTV8NotREVhis	acatgt**GCGGCCGC***TCA****ATGGTGATGGTGATGGTG***CATCATCACGAAACGCTTCTG	NotI	NS5 BTV-8
NS5BTV1EcoFor	gattct**GAATTC**ACGATGACACGATTTCCCAACC	EcoRI	NS5 BTV-1
NS5BTV1NotREV	acatgt**GCGGCCGC***TCA*CGTCATCACGAAACGCTTC	NotI	NS5 BTV-1
NS5BTV1NotREVHis	acatgt**GCGGCCGC***TCA****ATGGTGATGGTGATGGTG***CGTCATCACGAAACGCTTCTG	NotI	NS5 BTV-1
NS5AHSV4MarEcoFor	gattct**GAATTC**ATAATGGAGAGTCGGGGGCGAT	EcoRI	NS5 AHSV-4
NS5AHSV4MarNotRev	acatgt**GCGGCCGC***TTA*AAGTTCTGATACCCACCTGC	NotI	NS5 AHSV-4
NS5AHSV4MarNotRevHis	acatgt**GCGGCCGC***TTA****ATGGTGATGGTGATGGTG***AAGTTCTGATACCCACCTGC	NotI	NS5 AHSV-4
NS5KEMVEcoFor	gattct**GAATTC**GCAATGTCGCTCTTGGCGTTCTAC	EcoRI	NS5 KEMV
NS5KEMVNotRev	acatgt**GCGGCCGC***TCA*GTTTCGCCAGGGTGCGC	NotI	NS5 KEMV
NS5KEMVNotRevhis	acatgt**GCGGCCGC***TCA****ATGGTGATGGTGATGGTG***GTTTCGCCAGGGTGCGCTC	NotI	NS5 KEMV
eGFP-TAG_SapI	tatc**GCTCTTCA**CTACTTGTACAGCTCGTCCATGCC	SapI	eGFP
GS3G_eGFP-ATG_SapI	tatc**GCTCTTCA**GGTTCAGGCGGAGGGATGGTGAGCAAGGGCGAGGA	SapI	eGFP
pCI_NS5-fusion_For	tatc**GCTCTTCA**TAGGCGGCCGCTTCCCTTTAGTGA	SapI	pCI-NS5BTV1 (plasmid sequence)
pCI_NS5-fusion_Rev	tatc**GCTCTTCT**ACCCGTCATCACGAAACGCTTCTGCGT	SapI	pCI-NS5BTV1 (NS5-specific)
pCI-insSV40-ORF_Rev	tatc**GCTCTTCT**CATGGTGGCTCTAGCCTTAAGTTC	SapI	pCI-BTV1NS5-GFP (plasmid sequence)
pCI-insSV40-ORF_For	tatc**GCTCTTCA**TAGCGGGACTCTGGGGTTCGA	SapI	pCI-BTV1NS5-GFP (plasmid seqeunce)
BTV1VP5-ATG_SapI	tatc**GCTCTTCA**ATGGGTAAAGTCATACGGTCCTTAAG	SapI	VP5 BTV-1
BTV1VP5-TAG_SapI	tatc**GCTCTTCA**CTAAGCATTTCGTAAGAAGAGTGGTACGT	SapI	VP5 BTV-1

Table S1: primers used for cloning into bacterial (pGEX) or mammalian plasmids. Sequence in lower case letter represent a non-specific tail to ensure efficient digestion by restriction enzymes. Sequences in non-italic bold characters are those of restriction enzyme sites. Underlined sequences are target specific. Sequences in italic bold characters encode the 6xHis tag. Glycine/glycine-serine linkers are underlined with a double line. Stop codons are shown in red.

Primer	Sequence (5'→3')	Target
Seg10BTV-1_mut2xM-NS5-For	**ACG**ACACGAUUUCCCAACCGCCGAGAU**ACG**CU	mutating ATG codons in NS5 ORF
Seg10BTV-1_mut2xM-NS5-Rev	AGCGUAUCUCGGCGGUUGGGAAAUCGUGUCGU	mutating ATG codons in NS5 ORF
BTV_S10_F	TGGAYAAAGCRATGTCAAA	Seg-10 based real-time PCR assay
BTV_S10_R	ACRTCATCACGAAACGCTTC	BTV Seg-10 based real-time PCR assay
BTV_S10_P	FAM-ARGCTGCATTCGCATCGTACGC-BHQ1	BTV Seg-10 based real-time PCR assay
q1S1-set2F	GACCAGAACTTATCTCCGCAG	BTV-1 Seg-1 based real-time PCR assay
q1S1-set2Rbis	GCATAACGACATCCTTTCTC	BTV-1 Seg-1 based real-time PCR assay
q1S3-set2F	AGAGTCCAAGTCAATTATGGTC	BTV-1 Seg-3 based real-time PCR assay
q1S3-set2R	TGTAGCCCATCCATTATATCCT	BTV-1 Seg-3 based real-time PCR assay
q1S7-set1F	GGGTAACTCACAGCAAACTC	BTV-1 Seg-7 based real-time PCR assay
q1S7-set1R	AAGGCAGGGTATTGATTTAAGG	BTV-1 Seg-7 based real-time PCR assay
CoxIHamFor	GATTTGGAAACTGACTTGTAC	hamster's cytochrome oxidase I
CoxIHamRev	AGACTGTTCAACCAGTTCCAGC	hamster's cytochrome oxidase I

Table S2: deoxy-uracil containing primers used for mutating two ATG codons in the NS5 ORF of BTV-1 (ATG mutated to ACG), real-time PCR primers used to amplify viral RNAs derived from genome segments 1, 3, 7 or 10 of BTV and real-time PCR primers targeting hamster's cytochrome oxidase I gene.

Primer	Sequence (5'→3')	Target
IRESYFP-BsaI-F	tact**GGTCTCaCGAG**CATGCATCTAGGGCG	**ShuttleVacc**
ShuttleBsaI-R	tact**GGTCTCtTGGA**TCCGAATTCCTGCAGAT	**ShuttleVacc**
NS5BTV1BsaI-F	tact**GGTCTCgTCCA**AgGGATGACACGATTTCCCAA	**NS5**
NS5BTV1BsaI-R	tact**GGTCTCgCTCG**TCACGTCATCACGAAACGCTTCTG	**NS5**
E3LBsaI-F	tact**GGTCTCgTCCA**AACGATGTCTAAGATCTATATTGACG	**E3L**
E3LBsaI-R	tact**GGTCTCgCTCG**TCAGAATCTAATGATGACGTAAC	**E3L**

Table S3: primers used to insert NS5 or E3L ORFs into the ShuttleVacc plasmid. Sequence in lower case letter represent non-specific tail to ensure efficient digestion by BsaI restriction enzyme (sequence in bold characters). Underlined sequences are target specific.
